# Comprehensive metabolomics profiling reveals novel biomarkers and pathways for early detection of Alzheimer’s disease

**DOI:** 10.1093/braincomms/fcaf410

**Published:** 2025-10-18

**Authors:** Prabhakar Tiwari, Anu Gupta, Meenakshi Kaushik, Anjali Yadav, Anjali Anjali, Rekha Dwivedi, Pallavi Mudgal, Yashwant Kumar, Manjari Tripathi, Rima Dada

**Affiliations:** Department of Anatomy, All India Institute of Medical Sciences, New Delhi 110029, India; Department of Neurology, All India Institute of Medical Sciences, New Delhi 110029, India; Department of Anatomy, All India Institute of Medical Sciences, New Delhi 110029, India; Department of Anatomy, All India Institute of Medical Sciences, New Delhi 110029, India; Department of Neurology, All India Institute of Medical Sciences, New Delhi 110029, India; Department of Neurology, All India Institute of Medical Sciences, New Delhi 110029, India; Translational Health Science and Technology, NCR Biotech Science Cluster, Faridabad, Haryana 121004, India; Translational Health Science and Technology, NCR Biotech Science Cluster, Faridabad, Haryana 121004, India; Department of Neurology, All India Institute of Medical Sciences, New Delhi 110029, India; Department of Anatomy, All India Institute of Medical Sciences, New Delhi 110029, India

**Keywords:** Alzheimer's disease, plasma biomarkers, metabolomics, cognition, neurodegenerative disease

## Abstract

Alzheimer’s disease is a multifactorial neurodegenerative disorder marked by cognitive decline, synaptic dysfunction, and metabolic alterations. This study investigated disease-associated profiles in the Indian population using integrated clinical, metabolomic, and plasma biomarker analyses. We enrolled 25 clinically diagnosed patients (mean age: 61.20 ± 7.76 years) and 25 cognitively healthy controls (mean age: 60.56 ± 7.48 years). Cognitive and neuropsychiatric assessments included Addenbrooke’s Cognitive Examination-III, Clinical Dementia Rating-Global, and Patient Health Questionnaire-9 for patients, and Montreal Cognitive Assessment, Clinical Dementia Rating-Global, and Patient Health Questionnaire-9 for controls. Plasma metabolomics was performed using liquid chromatography–mass spectrometry, and targeted ELISA quantified amyloid beta 40, amyloid beta 42, phosphorylated tau181, phosphorylated tau217, neurofilament light chain, apolipoprotein E, APOE4, 8-hydroxy-2′-deoxyguanosine, C-reactive protein, brain-derived neurotrophic factor, and glutamate. Statistical analyses included principal component analysis, volcano plots, receiver operating characteristic curves, pathway enrichment, and correlation analyses. Patients showed reduced cognition (median Addenbrooke’s Cognitive Examination-III: 26). Clinical Dementia Rating-Global scores (1.44 ± 0.65 versus 0.24 ± 0.25; *P* < 0.0001) and Patient Health Questionnaire-9 scores (4.88 ± 4.21 versus 0.20 ± 0.50; *P* < 0.0001) were higher than controls. Principal component analysis revealed distinct metabolic clustering with 75 altered metabolites. Volcano analysis identified six upregulated (leucine, ascorbic acid, guanine) and 14 downregulated metabolites (valine, nicotinamide, octadecanedicarboxylic acid). Receiver operating characteristic curves highlighted octadecanedicarboxylic acid (AUC = 0.917), prolinamide (AUC = 0.908), 2-phosphoglycerate (AUC = 0.858), nicotinamide (AUC = 0.848), leucine (AUC = 0.768), and ascorbic acid (AUC = 0.748). Pathway enrichment indicated disruptions in branched-chain amino acid metabolism, nicotinamide metabolism, the tricarboxylic acid cycle, and neurotransmitter pathways. Biomarker analysis revealed elevated amyloid beta 40, amyloid beta 42/40 ratio, phosphorylated tau181, phosphorylated tau217, phosphorylated tau217/amyloid beta 42 ratio, neurofilament light chain, APOE4, C-reactive protein, and 8-hydroxy-2′-deoxyguanosine, with reduced brain-derived neurotrophic factor (all *P* < 0.05). Significant correlations included eupatilin with phosphorylated tau217 and 8-hydroxy-2′-deoxyguanosine, glyceraldehyde with brain-derived neurotrophic factor, guanine with APOE4, and valine inversely with phosphorylated tau181. This study identifies distinct metabolic (octadecanedicarboxylic acid, prolinamide, leucine, ascorbic acid) and biomarker profiles (phosphorylated tau217, 8-hydroxy-2′-deoxyguanosine, brain-derived neurotrophic factor) in Alzheimer’s disease. Disrupted pathways linked to neuroinflammation and oxidative stress support the potential for integrated early detection strategies. Despite the small cross-sectional cohort, findings highlight the need for longitudinal, multi-centric validation.

## Teaser

Tiwari *et al*. report that Indian Alzheimer’s disease patients show distinct plasma metabolite and biomarker profiles, including altered amino acid and nicotinamide pathways, elevated plasma phosphorylated tau217/amyloid beta42 ratio, and oxidative stress markers, highlighting population-specific signatures and supporting integrated biomarker–metabolomics approaches for early detection.

## Graphical Abstract

**Graphical Abstract:**
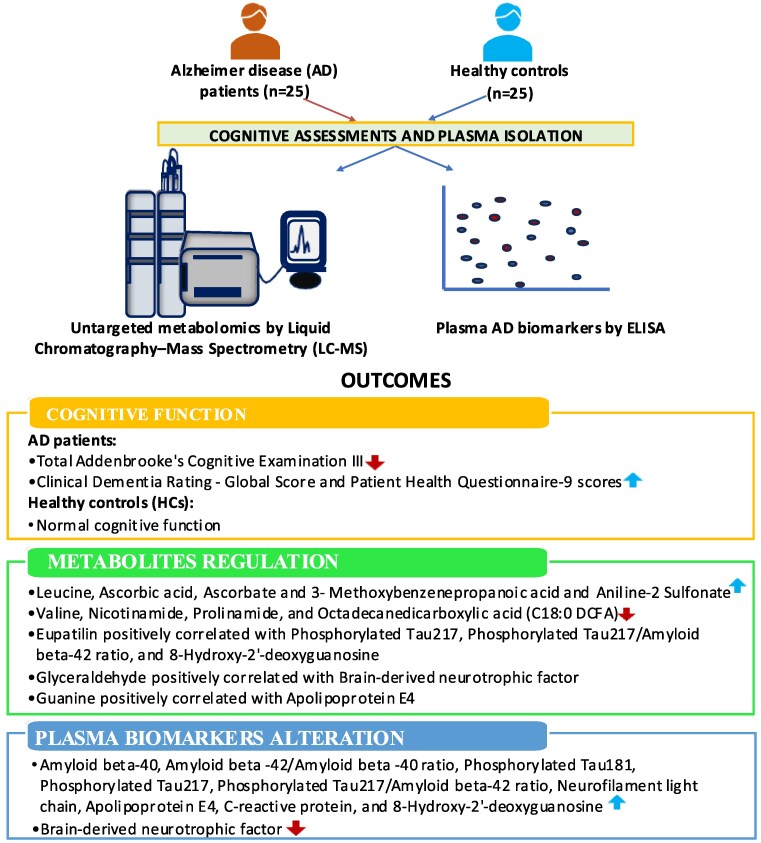

## Introduction

Alzheimer’s disease (AD) is a progressive neurodegenerative disorder and the leading cause of dementia globally, affecting over 55 million individuals in 2020 and projected to rise to 78 million by 2030.^1-3^ AD is characterized by the accumulation of extracellular amyloid-β (Aβ) plaques and intracellular neurofibrillary tangles composed of hyperphosphorylated tau, which collectively contribute to synaptic loss, neurodegeneration, and cognitive decline.^4^ Despite increasing prevalence, early and accurate diagnosis remains a significant clinical challenge, particularly due to overlapping presentations with other dementia subtypes such as vascular dementia (VaD), frontotemporal dementia (FTD), and dementia with Lewy bodies (DLB).^5^ While cerebrospinal fluid (CSF) assays and Amyloid-Positron Emission Tomography (amyloid-PET) imaging provide diagnostic specificity, their high cost and invasive nature limit their widespread use, emphasizing the need for reliable, accessible, and non-invasive biomarkers.^6^ Plasma biomarkers such as amyloid beta 40 (Aβ40), amyloid beta 42 (Aβ42), tau phosphorylated at threonine 181 (pTau181), tau phosphorylated at threonine 217 (pTau217), and neurofilament light chain (NFL) have shown strong correlations with core AD pathologies, including amyloid accumulation, tau hyperphosphorylation, and neuroaxonal injury.^5-8^ However, these markers alone may not fully capture the complexity of AD pathogenesis, which also involves mitochondrial dysfunction, oxidative stress, neuroinflammation, and metabolic dysregulation. Oxidative DNA damage, as indicated by elevated 8-hydroxy-2′-deoxyguanosine (8-OHdG), and impaired glutamate homeostasis have been implicated in neuronal loss and disease progression.^9-11^ Furthermore, genetic risk factors such as the apolipoprotein E ɛ4 (APOE4) allele exacerbate oxidative stress and metabolic vulnerability, thereby amplifying tau pathology and accelerating cognitive decline.^12^ Inflammatory markers such as C-reactive protein (CRP) and decreased levels of neurotrophic factors like brain-derived neurotrophic factor (BDNF) have also been associated with neurodegeneration and impaired synaptic plasticity.^13-15^ Despite these advancements, the diagnostic sensitivity and specificity of plasma biomarkers remain limited, particularly in early-stage and atypical presentations of AD.

Metabolomics, by capturing a comprehensive profile of small-molecule metabolites, provides an integrated snapshot of biochemical activity and cellular physiology, and serves as a powerful approach to elucidate the metabolic underpinnings of AD. Previous studies have identified disruptions in lipid metabolism, mitochondrial energy production, amino acid homeostasis, and redox balance in AD patients.^16-18^ However, current evidence is predominantly based on Western cohorts, with minimal representation from ethnically and genetically diverse populations. Furthermore, integrative approaches combining plasma biomarker analysis with comprehensive metabolomic profiling remain largely unexplored, limiting the ability to achieve a holistic understanding of AD pathophysiology. To address these gaps, we implemented a multi-omics strategy integrating untargeted metabolomic profiling using Liquid Chromatography–Mass Spectrometry (LC-MS) with quantitative plasma biomarker assessment using ELISA in an Indian cohort.

## Materials and methods

### Study design and participants recruitment

This case-control study was conducted in the Outpatient Department (OPD) of the Department of Neurology at the All India Institute of Medical Sciences (AIIMS), New Delhi, a hospital-based tertiary care center. A total of 50 participants were enrolled, including 25 clinically diagnosed Alzheimer’s disease (AD) patients and 25 age-matched healthy controls (HCs). All procedures were conducted in accordance with the Declaration of Helsinki. The study protocol was approved by the Institutional Ethics Committee, AIIMS New Delhi (IEC No. AIIMSA00394/12-01-2024; RP-13/2024), and written informed consent was obtained from all participants prior to enrolment. A detailed study flowchart is provided in Supplementary Fig. 1.

Participants were recruited according to predefined inclusion and exclusion criteria. Inclusion criteria for the AD group were: age 45–75 years, clinical diagnosis of AD, cognitive impairment confirmed by the Addenbrooke’s Cognitive Examination-III (ACE-III) and Clinical Dementia Rating-Global Score (CDR-G), and diagnosis validated using the National Institute on Aging-Alzheimer’s Association (NIA-AA) guidelines,^19^ supported by neuroimaging findings from brain magnetic resonance imaging (MRI) and fluorodeoxyglucose positron emission tomography (FDG-PET). HCs were screened using the Montreal Cognitive Assessment (MoCA) and CDR-G and confirmed the normal cognition.

Exclusion criteria included major psychiatric disorders (e.g. schizophrenia, bipolar disorder, major depressive disorder); neurological conditions other than AD (e.g. Parkinson’s disease, stroke, epilepsy, traumatic brain injury); vascular diseases; active malignancy; chronic kidney, liver, lung, or heart dysfunction; antibiotic use within the preceding three months; major gastrointestinal illness or surgery within the preceding year; or unwillingness to provide informed consent.

Comprehensive clinical profiling was carried out using structured case record forms to capture demographic information, lifestyle factors (smoking, alcohol use), family and educational history, and age at disease onset. Disease severity was assessed using the CDR-G, and depressive symptoms were evaluated with the Patient Health Questionnaire-9 (PHQ-9).

### Sample collection and processing

A total of 5 mL of whole blood was collected using EDTA tubes (Vacutainer® K2EDTA, BD Diagnostics), immediately centrifuged at 1500 g for 15 min at 4°C, and the plasma was aliquoted into 1.5 mL Eppendorf tubes and stored at −80°C until analysis. These samples were used for both untargeted metabolomics profiling using LC-MS and AD related-plasma biomarker analysis (Aβ40, Aβ42, pTau181, pTau217, NFL, BDNF, 8-OHdG, CRP, APOE, APOE4, and glutamate) using commercially available enzyme-linked immunosorbent assay (ELISA) kits.

### Metabolomics profiling using LC-MS

Plasma samples from AD patients and HCs were thawed on ice. A 100 µL aliquot of plasma was mixed with 100 µL of water containing 0.15% formic acid. Acetonitrile was added to the plasma at a 1:4 ratio to precipitate proteins. The mixture was vortexed, sonicated for 15 min at 1400 rpm and 4°C, and centrifuged at 14 000 rpm for 30 min at 4°C. The resulting supernatant was transferred to vials and analyzed using Ultra Performance Liquid Chromatography (UPLC) coupled with a time-of-flight mass spectrometer (ToF MS, SYNAPT G2, Waters Inc.). Analytes were separated on a 2.1 × 100 mm, 1.7-μm BEH amide column (Waters Inc.) maintained at 40°C, with mass spectrometry performed in positive electrospray ionization mode and corrected for lock mass deviations.

### Metabolomics data acquisition and processing using LC-MS

Metabolomics raw data were processed using *Progenesis QI* software, which facilitated retention time (RT) alignment, peak deconvolution, feature detection, and preliminary metabolite annotation. Metabolite identification was conducted via the *Metascope* plug-in, utilizing an in-house spectral library comprising approximately 600 reference compounds. Identification criteria included a spectral similarity threshold of ≥30% and RT matching within ±0.5 min. To ensure analytical robustness, features exhibiting a coefficient of variation (CV) > 30% in pooled quality control (QC) samples were excluded from downstream analysis.

### Plasma biomarkers profiling

Plasma levels of biomarkers including Aβ40, Aβ42, Aβ42/Aβ40 ratio, pTau181, pTau217, NFL, apolipoproteins (APOE, APOE4), glutamate, BDNF, CRP, and 8-OHdG were quantified using commercially available ELISA kits. Aβ40 and Aβ42 levels were measured using R&D Systems kits (DAB140B and DAB142), while pTau181 and glutamate were measured via Invitrogen assays (KHO0631 and EEL065). pTau217 was quantified with the MyBioSource ELISA kit (MBS1608795). Fine-Test kits (EU2548, EH0043, and EH0099) were used for detecting 8-OHdG, BDNF, and CRP, respectively. NFL levels were determined using Elabscience ELISA kit (E-EL-H6203), and APOE/APOE4 concentrations were measured using Krishgen Biosystems kits (KBH20167 and KBH4761). All assays were performed in duplicate following the respective manufacturers’ protocols. Absorbance was recorded at 450 nm using a calibrated microplate reader, and biomarker concentrations were extrapolated from standard curves, reported in pg/mL or ng/mL, as appropriate.

### Statistical analysis

Demographic and clinical variables were compared between AD and HCs groups using independent Student’s *t*-tests. Metabolomic data were processed and analyzed using MetaboAnalyst v6.0, including sum normalization, log transformation, and Pareto scaling to stabilize variance and reduce heteroscedasticity. Unsupervised Principal Component Analysis (PCA) was performed to assess sample clustering and overall data structure, with model quality evaluated by *R*² and *Q*² metrics.

Group-wise differences in metabolite abundance were assessed using Student’s *t*-tests, with statistical significance set at *P* < 0.05 and corrected for multiple comparisons using the false discovery rate (FDR). Metabolites were considered significantly dysregulated if they met all of the following criteria: fold change ≥2.0, Variable Importance in Projection (VIP) score >1.2 from partial least squares discriminant analysis (PLS-DA), and FDR-adjusted *P*-value <0.05. Volcano plots were generated to visualize metabolites meeting both statistical and biological thresholds, and hierarchical clustering with heatmaps was employed to explore expression patterns across samples. Pathway enrichment analysis of 20 significantly altered metabolites was performed using Over-Representation Analysis (ORA) via Metabolite Set Enrichment Analysis (MSEA) integrated with KEGG databases to identify biologically relevant metabolic pathways. The diagnostic performance of selected metabolites and plasma biomarkers was assessed using binary logistic regression and Receiver Operating Characteristic (ROC) curve analysis in MetaboAnalyst v6.0 and GraphPad Prism v8.0, respectively, reporting sensitivity, specificity, and area under the curve (AUC). Correlations among metabolites were calculated in MetaboAnalyst, while correlations between plasma biomarkers and between biomarkers and metabolites were evaluated using Spearman’s correlation tests in STATA v16.0. Heatmaps representing these correlations were generated in GraphPad Prism v8.0 to visualize coordinated changes.

## Results

### Demographic, clinical and cognitive outcome

The demographic and clinical characteristics of AD patients and HCs are summarized in Table 1. The mean age was comparable between groups (AD: 61.20 ± 7.76 years; HC: 60.56 ± 7.48 years), and the gender distribution did not significantly differ (AD: 13 males, 12 females; HC: 15 males, 10 females). The mean age at onset in the AD group was 57.12 ± 7.33 years. Educational attainment was lower in AD patients (10.00 ± 7.14 years) compared with HCs (12.20 ± 5.45 years), but this difference was not statistically significant. Cognitive performance, as measured by the CDR-G, was significantly higher in AD patients (1.44 ± 0.65) compared with controls (0.24 ± 0.25; *P* < 0.0001), indicating significant functional decline. The total ACE-III score was also reduced in the AD group, with a median of 26, indicating cognitive impairment. Depressive symptoms, assessed by the PHQ-9 score, were significantly higher in AD patients (4.88 ± 4.21) relative to HCs (0.20 ± 0.50; *P* < 0.0001). Lifestyle risk factors such as smoking and alcohol use were reported only in the AD group (5 smokers and 5 alcohol users). No participants reported a family history of AD. Collectively, these results highlight significant cognitive differences between both groups, with comparable age and education levels, underscoring the clinical relevance of these variables in further analyses.

**Table 1:** Demographic and clinical characteristics of AD versus HCs

Variable	AD (*n* = 25)	HCs (*n* = 25)	*P*-value
Age (years), Mean ± SD	61.20 ± 7.76	60.56 ± 7.48	0.768
Gender (Male/Female) (*n*)	13/12	15/10	—
Age at onset (years), Mean ± SD	57.12 ± 7.33	NA	—
Education (years), Mean ± SD	10.00 ± 7.14	12.20 ± 5.45	0.231
CDR-G score, Mean ± SD	1.44 ± 0.65	0.24 ± 0.25	<0.0001****
Total ACE-III, Median (Min-Max)	26 (4–77)	ND	—
Family history of AD/dementia (*n*)	0	NA	—
Smoking/Alcoholic status (*n*)	5/5	0 (0)/0 (0)	—
Depression—PHQ-9 score, Mean ± SD	4.88 ± 4.21	0.20 ± 0.50	<0.0001****

This table presents demographic and clinical characteristics of AD patients and HCs. Continuous variables are presented as mean ± SD or median (minimum, maximum), as appropriate. Categorical variables are expressed as counts. Between-group comparisons were performed using independent *t*-tests for continuous variables and chi-square or Fisher’s exact tests for categorical variables.

Abbreviations: ACE-III, Addenbrooke’s Cognitive Examination-III; CDR-G, Clinical Dementia Rating—Global Score; NA, not available; ND, not done; NS, non-significant; PHQ-9, Patient Health Questionnaire-9. Significance codes: ****P < 0.0001.

### Untargeted metabolomics profiling reveals significant metabolic alterations in AD

Metabolomic analysis was performed using *MetaboAnalyst v6.0* following rigorous data pre-processing, including exclusion of metabolites with >20% missing values and imputation using limit-of-detection (LoD) estimates. After normalization, log transformation, and Pareto scaling, a total of 530 metabolites were statistically evaluated between AD patients and HCs. Student’s *t*-tests followed by FDR correction (*P* < 0.05) identified 75 significantly altered metabolites, indicating widespread metabolic disruptions in AD. Among the most significantly dysregulated compounds were *C18:0 DC FA (Octadecanedicarboxylic acid)* (*P* = 5.12 × 10⁻⁸, FDR = 2.15 × 10⁻⁵), *Prolinamide* (*P* = 8.12 × 10⁻⁸, FDR = 2.15 × 10⁻⁵), and *Valine* (*P* = 3.43 × 10⁻⁶, FDR = 0.0005) suggesting robust statistical significance. Additionally, other metabolites included *2-Phosphoglycerate*, *2-Oxovaleric acid*, *Nicotinamide*, *Ethanolamine phosphate*, and *Glycerol-myristate*, each contributing to distinct metabolic profiles in AD. These metabolites spanned diverse biochemical classes, including amino acid derivatives, fatty acids, phospholipids, and energy metabolism intermediates (Supplementary Fig. 2A and Supplementary Table 1). The corresponding heatmap (Supplementary Fig. 2B) visualizes expression patterns of the top 50 metabolites, revealing distinct clustering between AD and HC samples. Notably, the altered metabolites exhibit consistent group-level shifts, reinforcing their potential as disease-associated metabolic signatures.

### PCA and volcano plot analysis reveal distinct metabolic signatures differentiating AD from HCs

**Figure 1:**
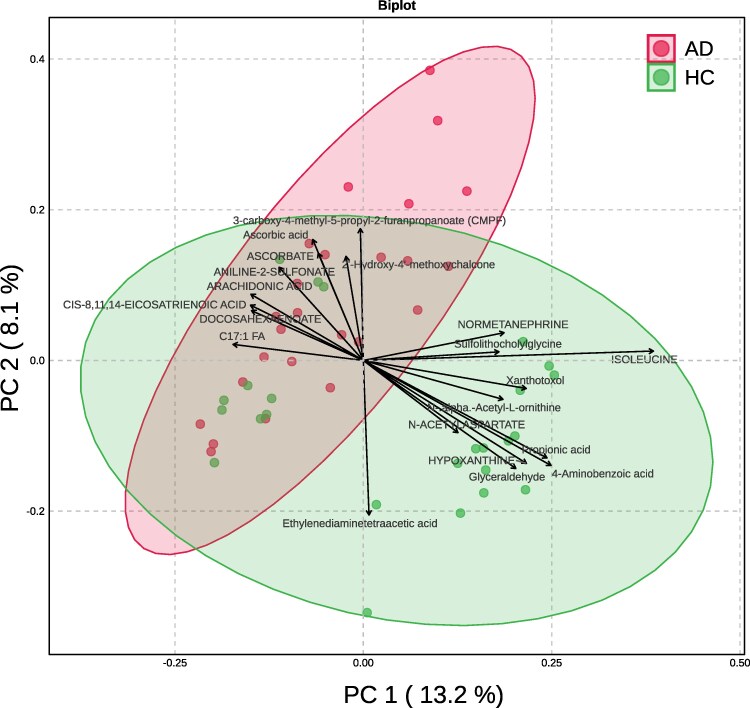
**PCA and volcano plot of metabolites identified by LC–MS: PCA of metabolites differentiating AD and HC:** PCA scores plot of metabolites, coloured according to diagnostic groups (red for AD and green for HC). Ellipses represent the 95% confidence interval for each group. PC1 and PC2 explain 13.13% and 8.08% of total variance, respectively. The biplot illustrates group separation based on metabolomic profiles. Analysis was performed using MetaboAnalyst (v6.0) software.

Metabolomic profiling revealed distinct biochemical signatures differentiating AD from HC, as demonstrated through integrative statistical analyses. Following quality control and LoD-based imputation, the normalized dataset was subjected to multivariate dimensionality reduction using PCA. PERMANOVA confirmed significant clustering between groups (*F* = 10.991; *R*² = 0.18632; *P* = 0.001), supporting metabolomic divergence at a systems level. The PCA bi-plot demonstrated clear separation of AD and HC cohorts along PC1 and PC2, explaining 13.2% and 8.1% of the total variance, respectively (Fig. 1).

**Figure 2:**
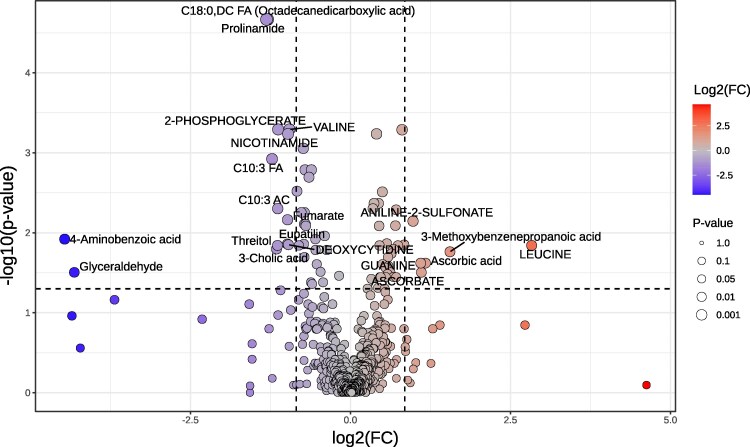
**Volcano plot highlighting significant metabolites in AD versus HC significantly downregulated = 14; significantly upregulated = 6; unsignificant = 510:** volcano plot of the metabolites identified by LC–MS, showing fold-change (Log2 scale, *x*-axis) versus significance (−Log10 *P*-value, *y*-axis). Vertical dotted lines indicate fold-change threshold (±1.8); horizontal dotted line denotes *P*-value threshold (0.05). Analysis conducted using MetaboAnalyst (v.6.0).

To complement multivariate insights, volcano plot analysis was performed using thresholds of FC-2.0 and *P* < 0.05, identifying metabolites with significant directional shifts in abundance. The most prominently upregulated metabolite in AD was Leucine (FC = 7.117; log₂(FC) = 2.831; *P* = 0.014), followed by 3-Methoxybenzenepropanoic acid, Ascorbic acid, ASCORBATE, Guanine, and Aniline-2-sulfonate all reflecting altered amino acid and redox-related pathways. Notably, several metabolites demonstrated pronounced downregulation including Valine, Nicotinamide, and Deoxycytidine, indicative of disrupted energy metabolism and nucleotide turnover. The strongest downregulated shifts were observed in C18:0 DC FA [Octadecanedicarboxylic acid, FC = 0.408; log₂(FC) = −1.294; *P* < 0.001], Prolinamide [FC = 0.403; log₂(FC) −1.311; *P* < 0.001], and Glyceraldehyde [FC = 0.050; log₂(FC) = −4.314; *P* = 0.031], implicating perturbations in dicarboxylic fatty acid oxidation and glycolytic flux (Fig. 2 and Table 2).

**Table 2:** Volcano plot analysis of differentially expressed plasma metabolites in AD versus HCs

Metabolites	FC	log2(FC)	*P*. adjusted	−Log10(*P* value)
Upregulated metabolites				
LEUCINE	7.117	2.831	0.014	1.842
3-Methoxybenzenepropanoic acid	2.939	1.555	0.017	1.763
Ascorbic acid	2.253	1.172	0.024	1.621
ASCORBATE	2.156	1.108	0.031	1.505
GUANINE	2.141	1.098	0.024	1.621
ANILINE-2-SULFONATE	1.970	0.978	0.007	2.145
Downregulated metabolites				
VALINE	0.517	−0.953	0.001	3.292
DEOXYCYTIDINE	0.511	−0.969	0.014	1.851
NICOTINAMIDE	0.509	−0.974	0.001	3.236
Fumarate	0.506	−0.982	0.007	2.163
Eupatilin	0.506	−0.984	0.014	1.859
2-PHOSPHOGLYCERATE	0.457	−1.130	0.001	3.292
C10:3 AC	0.455	−1.136	0.005	2.302
Threitol	0.454	−1.139	0.014	1.842
3-Cholic acid	0.449	−1.155	0.016	1.797
C10:3 FA	0.428	−1.225	0.001	2.922
C18:0,DC FA (Octadecanedicarboxylic acid)	0.408	−1.294	<0.001	4.667
Prolinamide	0.403	−1.311	<0.001	4.667
Glyceraldehyde	0.050	−4.314	0.031	1.505
4-Aminobenzoic acid	0.045	−4.465	0.012	1.921

This table presents plasma metabolites that were significantly altered between AD patients and age-matched HC, based on volcano plot analysis. For each metabolite, FC, log_2_-transformed FC, FDR-adjusted *P*-value, and −log_10_(*P*-value) are listed. Metabolites were considered differentially expressed if they met the criteria of *P* < 0.05 and absolute log_2_(FC) > 1. AC, Acylcarnitine; FA, Fatty acid; FC, Fold change.

### Metabolite-based ROC profiling identifies discriminatory biomarkers in AD

**Figure 3:**
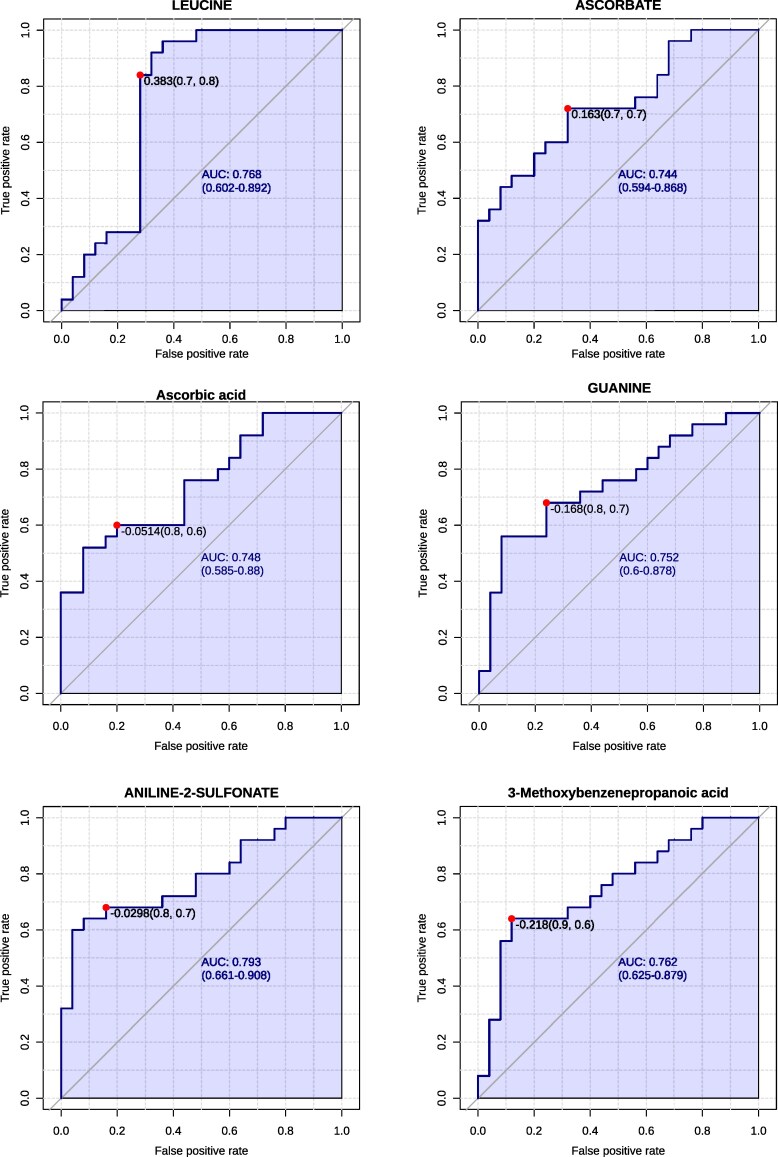
**Partial ROC curve of upregulated metabolic biomarkers.** Partial ROC curve showing the diagnostic performance of six upregulated metabolites distinguishing AD patients (*n* = 25) from HC (*n* = 25). The analysis demonstrates the sensitivity, specificity, and predictive accuracy of these metabolites as potential biomarkers.

**Figure 4:**
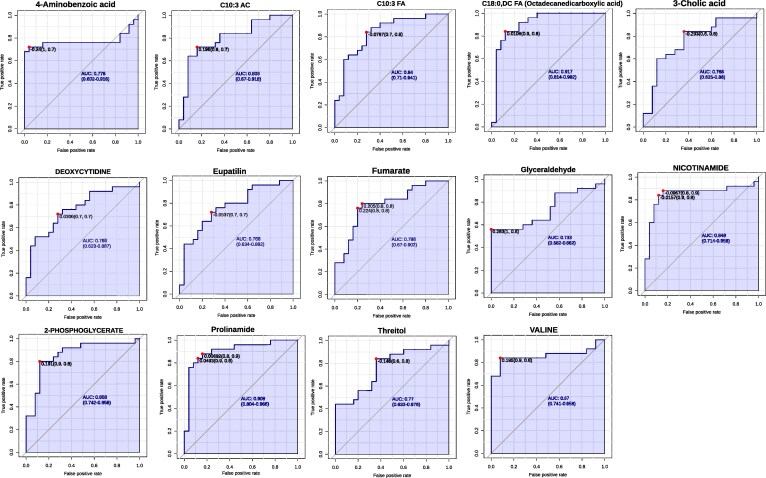
**Partial ROC curve of downregulated metabolic biomarkers.** Partial ROC curve showing the diagnostic performance of 14 downregulated metabolites differentiating AD patients (*n* = 25) from HC (*n* = 25). The analysis indicates their sensitivity, specificity, and predictive accuracy as potential biomarkers.

Partial ROC curve analysis, constrained to a false positive rate (FPR) ≤ 0.2, identified a panel of metabolites with strong discriminatory power between AD and HCs groups. This targeted approach emphasized true positive rates (TPR) and proximity to the optimal ROC point, thereby refining biomarker selection. Among the upregulated metabolites, Leucine [AUC = 0.768, *P* = 0.038, log₂(FC) = 2.478], 3-Methoxybenzenepropanoic acid [AUC = 0.762, *P* = 0.001, log₂(FC) = 1.464], and Ascorbic acid [AUC = 0.748, *P* = 0.001, log₂(FC) = 1.033] demonstrated robust classification performance. Guanine, Ascorbate, and Aniline-2-sulfonate also showed elevated levels in AD, with AUC values ranging from 0.74 to 0.79 and *P* < 0.01. In contrast, several downregulated metabolites exhibited stronger diagnostic potential. C18:0, DC FA [AUC = 0.917, *P* < 0.0001, log₂(FC) = –1.243] and Prolinamide [AUC = 0.908, *P* < 0.0001, log₂(FC) = –1.272], followed by Nicotinamide, C10:3 FA, and 2-Phosphoglycerate, each with AUC >0.83 and *P* < 0.01. Glyceraldehyde [AUC = 0.733, *P* < 0.0001, log₂(FC) = −4.294] and 4-Aminobenzoic acid [AUC = 0.776, *P* < 0.0001, log₂(FC) = −4.464] showed the most pronounced reductions. ROC curves for all upregulated and downregulated metabolites are presented in Figs 3 and 4, respectively, with full statistical metrics summarized in Table 3.

**Table 3:** ROC curve analysis of plasma metabolites differentiating AD versus HCs

Metabolites	AUC	*T*-tests	log2(FC)
Upregulated metabolites			
LEUCINE	0.768	0.038	2.478
3-Methoxybenzenepropanoic acid	0.762	0.001	1.464
Ascorbic acid	0.748	0.001	1.033
ASCORBATE	0.744	0.001	1.003
GUANINE	0.752	0.004	1.218
ANILINE-2-SULFONATE	0.793	7.919E−5	0.944
Downregulated metabolites			
VALINE	0.87	0.257	−0.898
DEOXYCYTIDINE	0.766	0.003	−1.001
** NICOTINAMIDE**	**0.848**	**5.9275E−5**	**−0.959**
Fumarate	0.798	4.7634E−4	−0.991
Eupatilin	0.768	6.8726E−4	−0.872
** 2-PHOSPHOGLYCERATE**	**0.858**	**0.003**	**−1.091**
C10:3 AC	0.806	3.6173E−4	−1.090
Threitol	0.77	9.7254E−4	−1.163
3-Cholic acid	0.766	0.0066489	−0.990
C10:3 FA	0.84	4.865E−5	−1.206
** C18:0, DC FA (Octadecanedicarboxylic acid)**	**0.917**	**2.0095E−8**	**−1.243**
** Prolinamide**	**0.908**	**1.2174E−7**	**−1.272**
Glyceraldehyde	0.733	3.1502E−4	−4.294
4-Aminobenzoic acid	0.776	8.6321E−6	−4.464

This table summarizes plasma metabolites distinguishing Alzheimer's disease (AD) patients from healthy controls (HC) based on ROC curve analysis. Columns show AUC (area under the ROC curve), *P*-values (FDR-adjusted, in standardized scientific notation), and log_2_ fold changes (log_2_FC). Positive log_2_FC indicates upregulation in AD; negative values indicate downregulation. Bolded metabolites indicate top candidates with AUC ≥ 0.85 and statistically significant *P*-values, highlighting strong diagnostic potential.

### Metabolic disruptions in AD unveiled through MSEA and KEGG pathway analysis

**Figure 5:**
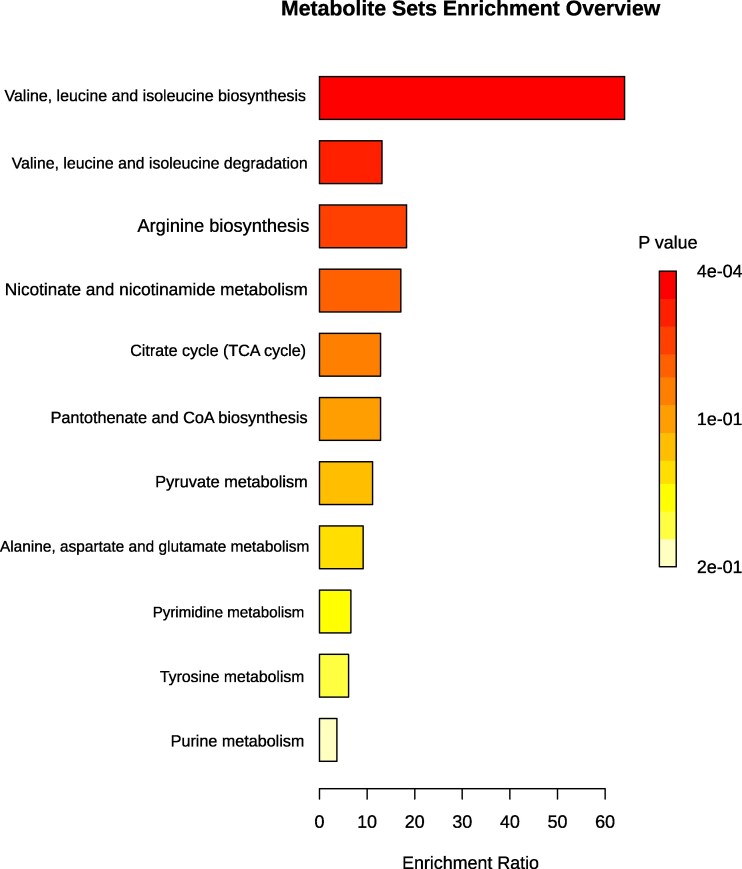
**Metabolite set enrichment and pathway analysis of differential metabolites in Alzheimer’s disease.** MSEA of differential metabolites: Pathway enrichment analysis, visualized as a bar graph, highlighting significantly altered metabolic themes in AD. Pathways are clustered into mitochondrial energy metabolism (e.g. citrate cycle), amino acid catabolism (e.g. valine, leucine, arginine metabolism), nucleotide metabolism (e.g. purine, pyrimidine biosynthesis), and neuroactive compound metabolism (e.g. nicotinate and tyrosine pathways). Bar lengths represent pathway enrichment scores, and asterisks denote statistically significant pathways based on FDR-adjusted *P*-values (<0.05), calculated using MetaboAnalyst software.

**Figure 6:**
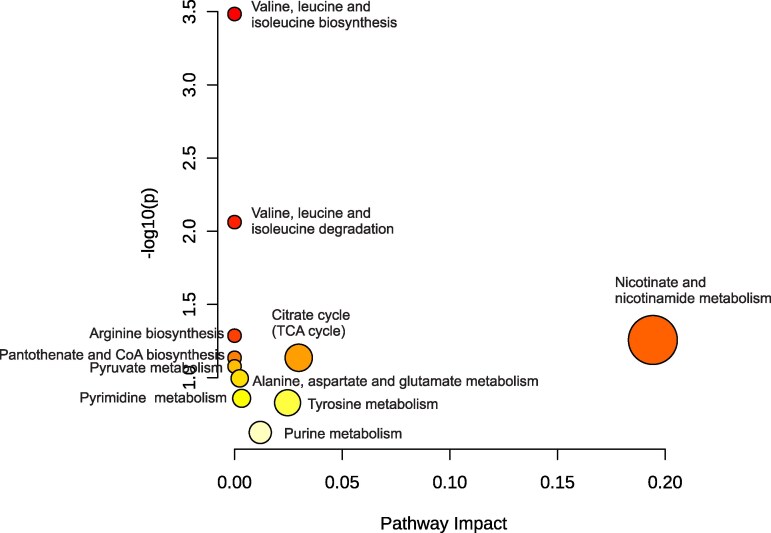
**KEGG pathway analysis of differential metabolites:** pathway impact analysis integrating metabolite abundance changes and pathway topology. The scatter plot displays impact scores (*x*-axis) against pathway significance (−log10 *P*-values, *y*-axis). Larger point size and darker shading reflect greater biological relevance. This visualization prioritizes key metabolic pathways implicated in AD pathophysiology. The analysis was performed using MetaboAnalyst software, combining quantitative metabolite data with pathway databases to uncover metabolic disruptions in AD.

Metabolic pathway enrichment and topology analyses revealed widespread dysregulation of biochemical networks in AD. Differentially abundant metabolites were analyzed using MetaboAnalyst v6.0 with MSEA and KEGG-based pathway impact scoring (Figs 5 and 6; Table 4). The most significantly affected pathways were those related to branched-chain amino acid (BCAA) metabolism. Valine, leucine, and isoleucine biosynthesis showed the strongest enrichment (MESA-*P* < 0.001, Holm *P* = 0.03, FDR = 0.03; KEGG- enrichment ratio ≈65, pathway impact ≈0.0, *P* < 0.001), while their degradation pathway was also significantly enriched (MESA: *P* = 0.01, Holm *P* = 0.70, FDR = 0.35; KEGG-enrichment ratio ≈14; pathway impact ≈0.0, *P* = 0.01), together indicating disruption of BCAA turnover that may impair neuronal protein homeostasis and neurotransmitter synthesis. Pathways related to mitochondrial and energy metabolism was also altered. Nicotinate and nicotinamide metabolism exhibited the highest pathway impact score (0.19) with strong enrichment (≈20), though statistical significance was modest (*P* = 0.06; Holm *P* = 1; FDR = 0.99 and 0.96) in both MESA and KEGG respectively, suggesting impaired NAD⁺ biosynthesis, redox balance, and mitochondrial function. Additional enrichment was observed in the citrate cycle (MESA-*P* = 0.08, Holm *P* = 1, FDR = 0.99; KEGG-enrichment ratio ≈14, pathway impact = 0.03, *P* = 0.07), pantothenate and CoA biosynthesis (MESA-*P* = 0.08, Holm *P* = 1, FDR = 0.99; KEGG-enrichment ratio ≈14, pathway impact = 0.0, *P* = 0.07), and pyruvate metabolism (MESA-*P* = 0.09, Holm *P* = 1, FDR = 0.99; KEGG-enrichment ratio ≈12, pathway impact = 0.0, *P* = 0.08), collectively pointing to reduced ATP generation and compromised oxidative phosphorylation. Dysregulation was also observed in amino acid pathways. Arginine biosynthesis was enriched (MESA-*P* = 0.05, Holm *P* = 1, FDR = 0.99; KEGG-enrichment ratio ≈20, pathway impact = 0.0, *P* = 0.05), suggesting disruption of nitric oxide signaling critical for neurovascular regulation, while alanine, aspartate, and glutamate metabolism (MESA-*P* = 0.10, Holm *P* = 1, FDR = 1; KEGG-enrichment ratio ≈10, pathway impact = 0.0, *P* = 0.10) indicated disturbances in excitatory and inhibitory neurotransmitter balance that may promote excitotoxicity. Perturbations in nucleotide metabolism were observed in pyrimidine (MESA-*P* = 0.14, Holm *P* = 1, FDS = 1.0; KEGG-enrichment ratio ≈8; pathway impact = 0.0, *P* = 0.14) and purine metabolism (MESA-*P* = 0.27; Holm *P* = 1, FDR = 1.0; KEGG-enrichment ratio ≈4, pathway impact = 0.01, *P* = 0.24), potentially reflecting impaired DNA/RNA synthesis and repair, thereby contributing to oxidative DNA damage and genomic instability. Finally, alterations in tyrosine metabolism (MESA-*P* = 0.16; Holm *P* = 1, FDR = 1.0; KEGG-enrichment ratio ≈6; pathway impact = 0.02, *P* = 0.15) suggested deficits in catecholamine biosynthesis, including dopamine and norepinephrine, further supporting neurotransmitter imbalance in AD.

**Table 4:** Pathway analysis of identified 20− metabolites using MESA and KEGG in AD

MESA matrix for 20 metabolites						
Metabolite set	Total	Hits	Expect	*P*-value	Holm *P*	FDR
Valine, leucine and isoleucine biosynthesis	8	2	0.03	3.51E−04	0.03	0.03
Valine, leucine and isoleucine degradation	39	2	0.15	0.01	0.70	0.35
Arginine biosynthesis	14	1	0.05	0.05	1.00	0.99
Nicotinate and nicotinamide metabolism	15	1	0.06	0.06	1.00	0.99
Pantothenate and CoA biosynthesis	20	1	0.08	0.08	1.00	0.99
Citrate cycle (TCA cycle)	20	1	0.08	0.08	1.00	0.99
Pyruvate metabolism	23	1	0.09	0.09	1.00	0.99
Alanine, aspartate and glutamate metabolism	28	1	0.11	0.10	1.00	1.00
Pyrimidine metabolism	39	1	0.15	0.14	1.00	1.00
Tyrosine metabolism	42	1	0.16	0.15	1.00	1.00
Purine metabolism	70	1	0.27	0.24	1.00	1.00

KEGG pathway matrix for 20 metabolites						
Pathway Name	*P*- value	−log(*P*)	Holm *P*	FDR	Impact	
Valine, leucine and isoleucine biosynthesis	3.28E−04	3.48	0.03	0.03	0.00	
Valine, leucine and isoleucine degradation	0.01	2.06	0.68	0.35	0.00	
Arginine biosynthesis	0.05	1.29	1.00	0.96	0.00	
Nicotinate and nicotinamide metabolism	0.06	1.26	1.00	0.96	0.19	
Pantothenate and CoA biosynthesis	0.07	1.14	1.00	0.96	0.00	
Citrate cycle (TCA cycle)	0.07	1.14	1.00	0.96	0.03	
Pyruvate metabolism	0.08	1.08	1.00	0.96	0.00	
Alanine, aspartate and glutamate metabolism	0.10	1.00	1.00	1.00	0.00	
Pyrimidine metabolism	0.14	0.86	1.00	1.00	0.00	
Tyrosine metabolism	0.15	0.83	1.00	1.00	0.02	
Purine metabolism	0.24	0.63	1.00	1.00	0.01	

This table presents the results of a metabolite set enrichment analysis (MESA) conducted on 20 selected metabolites to identify significantly associated metabolic pathways. Each row corresponds to a specific pathway, showing how many metabolites from the input set (‘Hits’) were found in that pathway out of the total number of metabolites known to be associated with it (‘Total’). The ‘Expect’ column indicates the number of hits that would be expected by chance alone, providing a baseline for comparison. The ‘*P*-value’ represents the probability of observing the given number of hits (or more) under the null hypothesis of random distribution. To correct for multiple hypothesis testing, two adjusted *P*-values are reported: The Holm-Bonferroni corrected *P*-value (‘Holm P’) and the False Discovery Rate (‘FDR’). A pathway is considered significantly enriched if these adjusted *P*-values fall below a standard threshold (commonly 0.05), indicating that the presence of the metabolites in the pathway is unlikely to be due to chance.

### Modular metabolite interactions reveal functional metabolic axes in AD

To delineate the crosstalk of metabolites within modular biochemical axes involved in AD pathogenesis, we performed a comprehensive Spearman rank correlation coefficient analysis, which revealed a constellation of tightly coupled inter-metabolite relationships (|*r*| ≥ 0.3, *P* ≤ 0.05) (Supplementary Fig. 3 and Supplementary Table 2). A lipid-centric module was defined by strong co-regulation between C10:3 FA and C10:3 AC (*r* = 0.77, *P* = 1.00 × 10^−10^), with both metabolites positively associated with C18:0 DC FA (*r* = 0.70, *P* = 1.83 × 10⁻⁸; *r* = 0.53, *P* = 7.39 × 10⁻⁵), fumarate (*r* = 0.52, *P* = 9.56 × 10⁻⁵; *r* = 0.41, *P* = 0.0028), and prolinamide (*r* = 0.44, *P* = 0.0014; *r* = 036, *P* = 0.009), suggesting coordinated regulation of lipid oxidation, mitochondrial energetics, and amino acid turnover. Prolinamide emerged as a key integrator across multiple pathways, correlating with C18:0 DC FA (*r* = 0.67, *P* = 1.28 × 10⁻⁷), nicotinamide (*r* = 0.58, *P* = 1.19 × 10⁻⁵), eupatilin (*r* = 0.54, *P* = 6.27 × 10⁻⁵), glyceraldehyde (*r* = 0.47, *P* = 6.20 × 10⁻⁴), and 4-aminobenzoic acid (*r* = 0.62, *P* = 1.91 × 10⁻⁶), linking amino acid metabolism with redox defense and xenobiotic response. Nicotinamide formed a tightly connected axis with fumarate (*r* = 0.61, *P* = 2.08 × 10⁻⁶), C18:0 DC FA (*r* = 0.56, *P* = 2.93 × 10⁻⁵), deoxycytidine (*r* = 0.62, *P* = 1.49 × 10⁻⁶), 4-aminobenzoic acid (*r* = 0.60, *P* = 4.73 × 10⁻⁶), and eupatilin (*r* = 0.58, *P* = 8.81 × 10⁻⁶), reflecting a coordinated NAD⁺ biosynthetic and nucleotide salvage module.

Deoxycytidine showed a strong correlation with threitol (*r* = 0.84, *P* = 3.84 × 10⁻¹⁴) and glyceraldehyde (*r* = 0.45, *P* = 0.00113), suggesting interplay between nucleotide turnover and sugar alcohol metabolism. Eupatilin also correlated with glyceraldehyde (*r* = 0.43, *P* = 0.00208), while glyceraldehyde and 4-aminobenzoic acid were linked (*r* = 0.47, *P* = 0.0005), indicating crosstalk between glycolytic intermediates and folate-dependent redox pathways.

The antioxidant pair ascorbate and ascorbic acid were tightly coupled (*r* = 0.91, *P* = 0.0000), and both negatively associated with fumarate (*r* = −0.54, *P* = 5.76 × 10⁻⁵; *r* = −0.51, *P* = 1.62 × 10⁻⁴) and nicotinamide (*r* = −0.49, *P* = 3.06 × 10⁻⁴; *r* = −0.49, *P* = 3.54 × 10⁻⁴), suggesting a reciprocal relationship between redox buffering and mitochondrial coenzyme biosynthesis. Aniline-2-sulfonate was positively correlated with ascorbic acid (*r* = 0.35, *P* = 0.0139), ascorbate (*r* = 0.33, *P* = 0.0213), and 3-methoxybenzenepropanoic acid (*r* = 0.29, *P* = 0.0436), and negatively associated with nicotinamide (*r* = −0.54, *P* = 4.98 × 10⁻⁵), C10:3 FA (*r* = −0.46, *P* = 0.00069), prolinamide (*r* = −0.44, *P* = 0.0013), and 4-aminobenzoic acid (*r* = −0.57, *P* = 1.29 × 10⁻⁵), implicating it in redox regulation and inverse control of NAD⁺ and folate-linked pathways. Additional associations included 2-phosphoglycerate with valine (*r* = 0.60, *P* = 4.41 × 10⁻⁶) and prolinamide (*r* = 0.56, *P* = 2.47 × 10⁻⁵), and valine with prolinamide (*r* = 0.64, *P* = 5.65 × 10⁻⁷) and 4-aminobenzoic acid (*r* = 0.50, *P* = 2.22 × 10⁻⁴), reinforcing the integration of glycolytic flux and branched-chain amino acid metabolism. Together, these correlations define a modular biochemical network encompassing lipid remodeling, mitochondrial energetics, nucleotide repair, antioxidant defense, and amino acid dynamics domains central to AD pathophysiology and biomarker prioritization.

### Plasma biomarker significantly correlated with AD pathology

**Figure 7:**
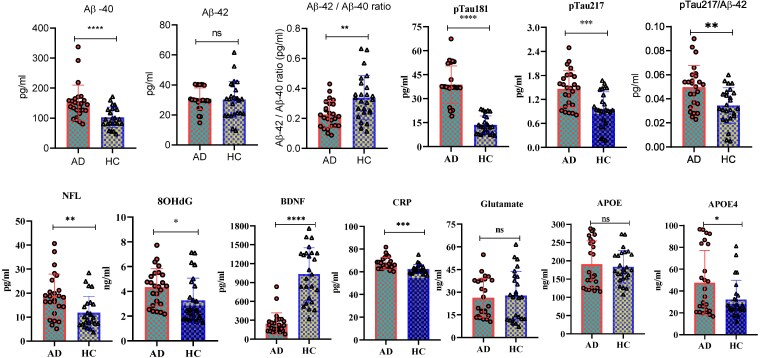
**Plasma levels of biomarkers in AD patients and HC from an Indian tertiary care center (*n* = 25 in each group):** bar plots representing group-wise mean concentrations (± standard deviation) of key plasma biomarkers associated with AD pathology, including amyloid-β isoforms (Aβ40, Aβ42), Aβ42/40 ratio, phosphorylated tau species (pTau181, pTau217), neurofilament light chain (NFL), 8-hydroxy-2′-deoxyguanosine (8-OHdG), brain-derived neurotrophic factor (BDNF), C-reactive protein (CRP), glutamate, apolipoprotein E (APOE), and APOE4 genotype status. All concentrations are standardized and reported in pg/mL or ng/mL, as appropriate (see Table 5). Statistical comparisons between AD and HC groups were conducted using two-tailed *t*-tests, with significance thresholds denoted as follows: *P < 0.05; **P < 0.01; ***P < 0.001; ***P < 0.0001. ‘ns’ indicates non-significant differences (*P* ≥ 0.05). Individual data points are overlaid to illustrate biological variability.

AD biomarkers level in plasma revealed significant differences in biomarker concentrations between AD patients and HCs, reflecting distinct pathophysiological processes. AD subjects exhibited elevated levels of Aβ40 (151.53 ± 58.48 pg/mL versus 94.86 ± 29.58 pg/mL; *P* < 0.0001), while Aβ42 levels were comparable across both groups (*P* = ns). However, the Aβ42/Aβ40 ratio was significantly reduced in AD (0.225 ± 0.088 versus 0.338 ± 0.021; *P* = 0.0019), suggesting impaired amyloid clearance. Tau pathology was reflected in elevated pTau181 (38.73 ± 11.67 pg/mL versus 13.68 ± 5.62 pg/mL; *P* < 0.0001) and pTau217 (1.46 ± 0.45 pg/mL versus 0.96 ± 0.43 pg/mL; *P* = 0.0002). Importantly, the pTau217/Aβ42 ratio, a clinically validated composite marker, was significantly higher in AD (0.0497 ± 0.024 versus 0.0345 ± 0.017; *P* = 0.0019), enhancing diagnostic insight. Markers of neurodegeneration showed consistent elevation in AD, including neurofilament light chain (NFL) (18.81 ± 9.06 pg/mL versus 11.85 ± 6.71 pg/mL; *P* = 0.0034) and APOE4 (47.54 ± 29.55 ng/mL versus 25.77 ± 10.42 ng/mL; *P* = 0.0011), while total APOE levels were not significant (*P* = ns), underscoring the relevance of isoform-specific profiling. BDNF levels were markedly reduced in AD (246.01 ± 170.15 pg/mL versus 1033.58 ± 419.61 pg/mL; *P* < 0.0001), indicating compromised neurotrophic effect. CRP was modestly elevated (67.43 ± 5.25 pg/mL versus 62.43 ± 4.01 pg/mL; *P* = 0.0004), consistent with low-grade systemic inflammation. Oxidative stress was reflected by increased 8OHdG (4.32 ± 1.50 ng/mL versus 3.27 ± 1.80 ng/mL; *P* = 0.0294). Glutamate levels did not differ significantly (*P* = ns), suggesting limited peripheral reflection of excitotoxicity. Group-wise graphical representations for AD versus HCs are illustrated in Fig. 7, and corresponding plasma levels are summarized in Table 5.

**Table 5:** Plasma biomarker levels in AD versus HCs

Biomarker	AD (*n* = 25), Mean ± SD	HC (*n* = 25) Mean ± SD	*P*-value
Aβ40 (pg/mL)	151.53 ± 58.48	94.86 ± 29.58	<0.0001****
Aβ42 (pg/mL)	30.77 ± 7.24	30.23 ± 12.10	ns
Aβ42/Aβ40 Ratio (pg/pg)	0.225 ± 0.088	0.338 ± 0.021	0.0019**
pTau217/Aβ42 Ratio (pg/pg)	0.0497 ± 0.024	0.0345 ± 0.017	0.0019**
pTau181 (pg/mL)	38.73 ± 11.67	13.68 ± 5.62	<0.0001****
pTau217 (pg/mL)	1.46 ± 0.45	0.96 ± 0.43	0.0002***
NFL (pg/mL)	18.81 ± 9.06	11.85 ± 6.71	0.0034**
APOE (ng/mL)	190.59 ± 63.92	184.00 ± 60.14	ns
APOE4 (ng/mL)	47.54 ± 29.55	25.77 ± 10.42	0.0011***
Glutamate (ng/mL)	26.32 ± 12.82	27.75 ± 16.09	ns
BDNF (pg/mL)	246.01 ± 170.15	1033.58 ± 419.61	<0.0001****
CRP (pg/mL)	67.43 ± 5.25	62.43 ± 4.01	0.0004***
8OHdG (ng/mL)	4.32 ± 1.50	3.27 ± 1.80	0.0294*

This table represents plasma concentrations of AD-relevant biomarkers measured in AD patients and HCs, represented as mean ± SD. Statistical comparisons were performed using two-tailed tests, and corresponding *P*-values indicate the level of significance. Biomarkers include amyloid beta peptides (Aβ40, Aβ42, and Aβ42/Aβ40 ratio), phosphorylated tau isoforms (pTau181, pTau217), NFL, apolipoprotein E species (APOE, APOE4), brain-derived neurotrophic factor (BDNF), C-reactive protein (CRP), and the oxidative DNA damage marker 8-hydroxy-2-deoxyguanosine (8OHdG). The pTau217/Aβ42 ratio is included as a composite diagnostic metric reflecting tau-to-amyloid burden. Significance indicators: *P* < 0.05 (*), *P* < 0.01 (**), *P* < 0.001 (***), *P* < 0.0001 (****), ns, not significant.

### Integrated ROC and correlation analysis identifies mechanistic biomarker axes in AD

To assess the diagnostic and mechanistic relevance of plasma biomarkers in AD, we performed ROC curve analysis alongside Spearman rank correlation coefficient profiling. Among amyloid markers, Aβ40 showed moderate diagnostic accuracy (AUC = 0.7840; *P* = 0.0006) and was significantly negatively correlated with the Aβ42/40 ratio (*r* = −0.63; *P* = 0.001), consistent with impaired clearance dynamics. Aβ42 failed to discriminate AD from HCs (AUC = 0.5216; *P* = 0.7934) and showed weak correlations with pTau217 (*r* = 0.18; *P* = 0.383) and 8OHdG (*r* = 0.18; *P* = 0.383). The Aβ42/40 ratio enhanced classification (AUC = 0.7512; *P* = 0.0023) and was positively associated with Aβ42 (*r* = 0.74; *P* = 0.0000). Tau biomarkers demonstrated high diagnostic value and pTau181 yielded near-perfect performance (AUC = 0.9784; *P* < 0.0001) but showed significant negative correlations with glutamate (*r* = −0.48; *P* = 0.015), while pTau217 (AUC = 0.7632; *P* = 0.0014) was positively correlated with glutamate but not significant (*r* = 0.31; *P* = 0.133) and strongly positively correlated with 8OHdG (*r* = 1.00; *P* = 0.0000), suggesting tau–oxidative and tau–excitotoxic coupling. The pTau217/Aβ42 ratio (AUC = 0.7344; *P* = 0.0045) also correlated with glutamate (*r* = 0.51; *P* = 0.0093) and 8OHdG (*r* = 0.68; *P* = 0.0002), reinforcing its integrative role in tau–amyloid burden and oxidative stress. Neurodegeneration and stress markers showed strong classification performance: BDNF (AUC = 0.9760; *P* < 0.0001) was not significantly correlated with other biomarkers, while NFL (AUC = 0.7480; *P* = 0.0026) showed a weak association with Aβ42 (*r* = 0.29; *P* = 0.167). CRP (AUC = 0.8040; *P* = 0.0002) correlated moderately with APOE4 (*r* = 0.16; *P* = 0.458), and 8OHdG (AUC = 0.7032; *P* = 0.0137) was significantly linked to pTau217 and glutamate, supporting oxidative stress as a downstream consequence of tau pathology. Genetic markers revealed mixed utility: APOE (AUC = 0.5136; *P* = 0.8690) showed no significant correlations, while APOE4 (AUC = 0.6304; *P* = 0.1138) was positively associated with Aβ40 (*r* = 0.25; *P* = 0.220). The ROC curves for AD biomarkers (Supplementary Fig. 4A), the Spearman *r* correlation heatmap (Supplementary Fig. 4B), and the Spearman rank *P*-values (Supplementary Table 3) summarize the relationships between the identified biomarkers for AD.

### Metabolite-biomarker correlations uncover distinct pathways and pathogenic mechanisms in AD

Spearman correlation analysis revealed several statistically significant associations (*P* ≤ 0.05) between plasma metabolites and AD-related biomarkers, highlighting distinct biochemical pathways implicated in disease pathogenesis. Among amyloid-related markers, C10:3 AC (acylcarnitine) showed a moderate positive correlation with Aβ42 (*r* = 0.42, *P* = 0.037), implicating medium-chain fatty acid metabolism in amyloidogenic processes. The Aβ42/40 ratio was inversely associated with prolinamide (*r* = −0.40, *P* = 0.049), while nicotinamide showed a borderline positive correlation (*r* = 0.35, *P* = 0.089), suggesting differential roles of amino acid derivatives in isoform-specific amyloid regulation.

Tau pathology exhibited strong associations with xenobiotic and oxidative stress markers. Both eupatilin, a flavonoid compound, and 8-hydroxy-2′-deoxyguanosine (8OHDG), a marker of oxidative DNA damage, were positively correlated with pTau217 (*r* = 0.53, *P* = 0.006) and the pTau217/Aβ42 ratio (*r* = 0.57, *P* = 0.003), highlighting a potential link between tau phosphorylation and oxidative stress or xenobiotic metabolism.

**Figure 8:**
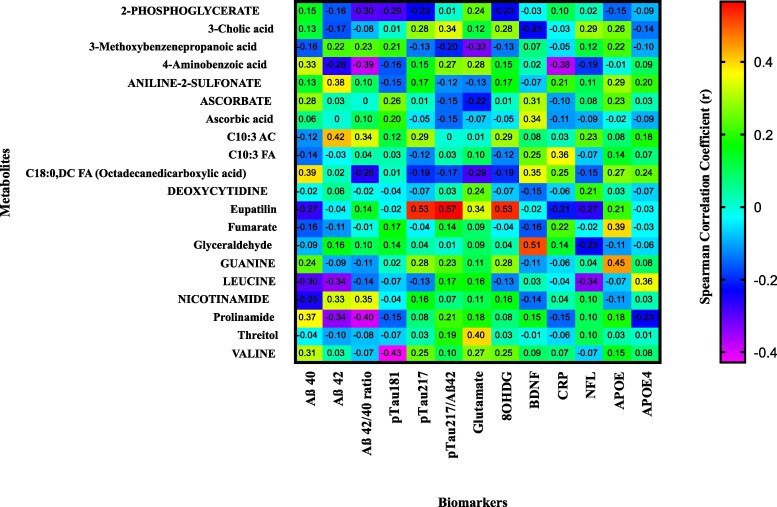
**Correlation heatmap between plasma biomarkers and identified metabolites in AD.** Correlation heatmap between plasma biomarkers and identified metabolites in AD (*n* = 25). The heatmap visualizes Pearson correlation coefficients between active metabolites (*y*-axis) identified through untargeted metabolomic profiling and plasma biomarkers (*x*-axis) associated with AD. Biomarkers include Aβ40, Aβ42, Aβ42/40 ratio, phosphorylated tau species (pTau181, pTau217, pTau217/Aβ42), NFL, 8-hydroxy-2′-deoxyguanosine (8-OHdG), brain-derived neurotrophic factor (BDNF), glutamate, CRP, apolipoprotein E (APOE), and APOE4 genotype. Metabolites include amino acids, fatty acids, organic acids, and oxidative stress markers relevant to AD pathophysiology. Colours represent Spearman correlation coefficients ranging from −0.4 (strong negative correlation; dark blue) to +0.4 (strong positive correlation; dark red), with the colour intensity proportional to the correlation magnitude. Positive correlations are shown in warm colours (yellow to red), while negative correlations are represented by cool colours (light blue to deep blue). This integrative correlation matrix highlights metabolite–biomarker associations that may reflect underlying molecular interactions and pathophysiological mechanisms in AD.

Neurotransmitter and neurotrophic pathways were also reflected in the metabolomic profile. Threitol, a sugar alcohol, correlated positively with glutamate levels (*r* = 0.40, *P* = 0.049), suggesting altered polyol metabolism may influence excitatory neurotransmission. Additionally, glyceraldehyde was positively associated with BDNF (*r* = 0.51, *P* = 0.01), indicating a potential role for glycolytic intermediates in neurotrophic signaling. Genetic risk was mirrored in purine metabolism, with guanine showing a strong positive correlation with APOE (*r* = 0.45, *P* = 0.025). A borderline association was also observed between fumarate and APOE (*r* = 0.39, *P* = 0.05), suggesting that TCA cycle intermediates may be modulated by APOE status. Finally, valine, a branched-chain amino acid, was inversely correlated with pTau181 (*r* = −0.43, *P* = 0.033), supporting a protective role of BCAA metabolism in tau pathology. These findings underscore a multifaceted biochemical landscape in AD, integrating lipid oxidation, amino acid turnover, oxidative stress, and genetic susceptibility, and highlight potential metabolomic targets for population-specific biomarker development. (Figure 8 and Supplementary Table 4).

## Discussion

Our study integrates untargeted metabolomics with cognitive, clinical, and plasma biomarker profiling to characterize AD patient. Our untargeted metabolomic profile reveals distinct metabolic alterations particularly in amino acid, mitochondrial, and nucleotide pathways that align with core AD mechanisms such as mitochondrial dysfunction, oxidative stress, and disrupted neuronal homeostasis.

This study provides an integrative assessment of demographic, clinical, cognitive, and biochemical parameters in AD patients versus HC within Indian cohort, providing population-specific insights into AD pathophysiology. The mean age was comparable between groups, consistent with previous findings from non-Western populations suggesting a somewhat earlier onset of sporadic AD, likely influenced by unique gene–environment interaction.^20,21^ The absence of family history across all AD cases supports a sporadic etiology, emphasizing the role of modifiable risk factors in disease progression.^22,23^ Educational attainment was lower in AD patients, aligning with the cognitive reserve hypothesis that links reduced education to increased dementia risk.^24,25^ Cognitive impairment was pronounced in the AD group, reflected by significantly higher CDR-G scores and reduced ACE-III performance, consistent with moderate-to-severe functional decline. Elevated PHQ-9 scores in AD patients further underscore the common co-occurrence of depression, potentially driven by shared neuroinflammatory and endocrine pathways.^26,27^ Lifestyle risk factors including smoking and alcohol use were reported in the AD group, resonating with previous findings that associate these exposures with oxidative stress and cerebrovascular dysfunction.^28-30^ Collectively, these results reinforce AD’s multifactorial nature and highlight how demographic alignment enhances the interpretability of metabolic and biomarker data in an Indian cohort.

### Metabolic dysregulation and biomarker identification in AD

Our untargeted plasma metabolomics profiling revealed 75 significantly altered metabolites in AD compared with HCs, highlighting systemic disturbances beyond amyloid- and tau-centric models. These dysregulated metabolites spanned amino acid catabolism, lipid metabolism, mitochondrial pathways, oxidative stress regulation, and redox homeostasis, underscoring the widespread metabolic dysregulation.^17,31^ A significant finding was the upregulation of Leucine a branched-chain amino acid (BCAA) known to activate the mammalian target of rapamycin (mTOR) pathway. Aberrant mTOR signaling is strongly implicated in impaired autophagy, lysosomal dysfunction, and protein aggregation in AD brains.^32,33^ Consistent with previous studies showing elevated BCAAs in plasma and CSF of AD patients,^31,34^ our data support the hypothesis that dysregulated BCAA metabolism exacerbates neuroinflammation, oxidative stress, and excitotoxic cascades, ultimately accelerating neurodegeneration. We also observed elevated plasma ascorbic acid levels in AD patients. This contrasts with consistent CSF reports of ascorbate deficiency in AD, attributed to impaired blood–brain barrier transport and increased central oxidative burden. The elevated plasma levels may reflect a compensatory peripheral antioxidant response to systemic oxidative stress. Importantly, such patterns may be influenced by regional dietary practices, as populations in India typically consume plant-rich, vitamin C–rich diets.^35,36^ These findings underscore the importance of contextualizing peripheral biomarkers within population-specific nutritional and metabolic baselines. Conversely, a key downregulated metabolite was C18:0 dicarboxylic fatty acid (DC FA), an intermediate in mitochondrial β-oxidation. Reduced levels likely reflect impaired lipid catabolism and mitochondrial energy failure, both hallmarks of AD pathology.^37,38^ The strong diagnostic accuracy of C18:0 DC FA is consistent with reports from Alzheimer’s Disease Neuroimaging Initiative (ADNI) and other Western cohorts, supporting its reliability as a cross-population biomarker.^39^ Additional amino acid perturbations further highlight widespread metabolic imbalance. Reduced Valine levels may compromise energy metabolism and neurotransmitter synthesis,^40^ while depletion of Prolinamide suggests disrupted glutamate cycling and redox buffering. Although not previously characterized in AD, reduced Prolinamide may represent a novel metabolic signature with links to non-canonical amino acid pathways.^41^ Another critical observation was reduced Nicotinamide, a key precursor of NAD⁺. NAD⁺ depletion is increasingly recognized as central to mitochondrial dysfunction, defective DNA repair, and impaired sirtuin activity in AD.^42,43^ Our enrichment analysis confirmed significant disruption of nicotinate and nicotinamide metabolism, supporting emerging therapeutic strategies aimed at NAD⁺ augmentation. Together, these findings align with growing evidence that NAD⁺-targeted interventions may hold therapeutic potential in AD. Multivariate PCA and PERMANOVA analysis (*P* = 0.001) demonstrated clear biochemical stratification between AD and HCs. ROC profiling highlighted Leucine (AUC = 0.768), Acorbic acid (AUC = 0.748), C18:0 DC FA (AUC = 0.917), 2-Phosphoglycerate (AUC = 0.859), Nicotinamide (AUC = 0.848) and Prolinamide (AUC = 0.908) as potential diagnostic markers.^31^ Pathway enrichment further revealed dysregulation in BCAA degradation, TCA cycle, arginine and proline metabolism, and nucleotide metabolism. These perturbations converge on mitochondrial dysfunction, energy failure, and impaired cellular maintenance, consistent with earlier metabolomic analyses of AD.^31,37^ Importantly, our metabolic cross-correlation network analysis revealed biochemical interconnectivity between redox regulation, lipid turnover, and mitochondrial pathways. Positive correlations, such as between plasma ascorbate and 3-methoxybenzenepropanoic acid, and inverse correlations between nicotinamide and C18:0 DC FA, suggest tightly regulated compensatory or co-pathological processes underlying AD progression. Compared with previous studies predominantly conducted in Western cohorts such as the ADNI and the Australian Imaging, Biomarkers and Lifestyle (AIBL) study, our study highlights both conserved and population-specific features.^44,45^ Conserved patterns include BCAA upregulation and NAD⁺ depletion, while unique findings such as elevated plasma ascorbate may reflect Indian dietary practices or genetic diversity. Such population-level heterogeneity highlights the importance of expanding biomarker discovery efforts across global cohorts to capture region-specific metabolic and environmental influences. Overall, our study reveals an integrated network of metabolic dysfunction in AD, with key nodes involving BCAA metabolism, nicotinamide/NAD⁺ homeostasis, mitochondrial lipid catabolism, and redox regulation. These findings not only reinforce established pathogenic pathways but also provide strong diagnostic potential metabolites biomarkers.

### Metabolite-biomarker networks reveal pathophysiological pathways in AD

Plasma biomarker profiling recapitulated hallmark AD processes including β-amyloid deposition, tau hyperphosphorylation, oxidative stress, neuroinflammatory activation, and neurodegeneration while revealing metabolite–biomarker correlations that advance mechanistic understanding of AD pathogenesis. Consistent with the amyloid cascade hypothesis, AD patients showed elevated plasma Aβ40 with unchanged Aβ42, yielding a significantly reduced Aβ42/40 ratio a robust marker of cerebral amyloidosis in Western studies.^46,47^ In our cohort, the reduced ratio demonstrated moderate diagnostic accuracy (AUC = 0.7512). Aβ42 alone did not discriminate AD from HCs, consistent with prior evidence of limited diagnostic value.^48,49^ Aβ40 correlated negatively with the Aβ42/40 ratio and positively with metabolites such as 2-phosphoglycerate and 4-aminobenzoic acid, suggesting that glycolytic and folate metabolism modulate amyloidogenic dynamics. Tau biomarkers showed stronger diagnostic performance. Plasma pTau181 and pTau217 were markedly elevated, with pTau181 achieving near-perfect accuracy (AUC = 0.9784). The pTau217/Aβ42 ratio further improved discrimination and correlated with oxidative DNA damage (8OHdG) and glutamate, implicating oxidative–excitotoxic cascades in tau pathology. These findings align with reports linking ROS to tau misfolding,^50,51^while a significant positive associations with eupatilin and guanine implicate xenobiotic and purine metabolism in tau phosphorylation. Markers of neurodegeneration reinforced disease mechanisms. Neurofilament light chain (NFL) and APOE4 were elevated, consistent with axonal injury and genetic vulnerability. APOE4 showed modest associations with Aβ40 and CRP, reflecting its role in lipid metabolism and neuroinflammation.^52^ BDNF was profoundly reduced, supporting evidence that impaired neurotrophic signaling contributes to synaptic dysfunction.^53,54^ Systemic inflammation and oxidative stress were reflected by elevated CRP and 8OHdG levels, pointing to peripheral drivers of neurodegeneration. 8OHdG showed strong correlations with pTau 217 (AUC-1.0) suggesting that oxidative DNA damage may amplify excitotoxic cascades a hypothesis supported by recent work implicating mitochondrial DNA damage in tau propagation.^9,55,56^ Metabolite associations revealed novel links between energy metabolism, amyloid processing, and tau pathology. Medium-chain acylcarnitine C10:3 correlated with Aβ42, implicating fatty acid metabolism in amyloidogenic processing. The Aβ42/40 ratio correlated inversely with Prolinamide but positively with Nicotinamide, indicating divergent roles of amino acid derivatives in amyloid regulation. Similarly, 2-phosphoglycerate correlated negatively with Aβ42 and the Aβ42/40 ratio, consistent with impaired glycolytic flux in AD.^57^ Tau pathology was also associated with oxidative and xenobiotic stress. Eupatilin and 8OHdG correlated with pTau217 and the pTau217/Aβ42 ratio, linking flavonoid signaling and oxidative DNA damage to tau phosphorylation.^58^ In parallel, 4-aminobenzoic acid positively correlated with both Aβ40 and pTau217, implicating folate-dependent one-carbon metabolism and MTHFR polymorphisms in disease risk.^59-61^ Nicotinamide correlated positively with the Aβ42/40 ratio, extending experimental evidence that NAD⁺ replenishment reduces amyloid burden and improves cognition.^62-65^ Diet- and microbiome-derived metabolites further shaped biomarker associations. Ascorbic acid correlated positively with BDNF and inversely with Aβ and tau markers, consistent with its antioxidant role. Higher ascorbate levels compared with Western cohorts likely reflect citrus- and green-rich diets^66,67^ whereas deficiency is linked to cognitive declines.^68^ Similarly, 3-methoxybenzenepropanoic acid correlated negatively with Aβ40 and positively with Aβ42, suggesting a protective isoform shift mediated by polyphenol.^69^ Neurotransmitter and neurotrophic pathways were also reflected in metabolite correlations. Threitol correlated positively with glutamate, implicating polyol metabolism in excitatory neurotransmission, while glyceraldehyde correlated with BDNF, linking glycolytic intermediates to neurotrophic signaling. Genetic susceptibility was reflected in purine and mitochondrial metabolism: guanine correlated with APOE and pTau217, implicating purine turnover in both genetic risk and tau aggregation, while fumarate correlated with APOE, linking TCA cycle intermediates with genetic vulnerability.

Interestingly, BCAAs showed evidence of protective effects. Valine correlated negatively with pTau181, suggesting a mitigating role against tau phosphorylation, while Leucine showed negative trends with amyloid ratios, consistent with reports that BCAAs regulate neurotransmitter synthesis, glutamate excitotoxicity, and mTOR signaling to influence synaptic plasticity and neuronal survival.^70,71^ In summary, our findings delineate a multifaceted biochemical landscape in AD that integrates fatty acid oxidation, glycolysis, one-carbon metabolism, oxidative stress, purine turnover, dietary influences, and genetic susceptibility. Population-specific features such as elevated ascorbate and polyphenol metabolites highlight dietary and microbiome contributions distinct from Western cohorts. Collectively, this integrative metabolite–biomarker framework advances mechanistic understanding of AD and identifies novel candidate biomarkers, reinforcing the need for population-specific approaches in biomarker discovery.

## Limitations

This study has several limitations. First, the relatively small sample size reduces statistical power and limits generalizability. Second, the cross-sectional design precludes causal inference and prevents evaluation of predictive performance over time. Third, reliance on plasma biomarkers and metabolite profiling without complementary cerebrospinal fluid (CSF) data may introduce diagnostic heterogeneity and restrict the ability to capture central nervous system (CNS)-specific alterations. Direct comparison of plasma and CSF profiles would be necessary to validate and strengthen the biological relevance of these findings.

Plasma metabolite levels may also be influenced by confounding factors such as diet, medications, and comorbidities, with some alterations reflecting systemic rather than CNS-driven processes. The observed metabolic signatures may therefore represent a combination of AD pathology and population-specific influences. For example, Indian dietary patterns—rich in flavonoids, polyphenols, and antioxidant vitamins (e.g. turmeric, citrus fruits, leafy greens) could contribute to elevated protective metabolites such as eupatilin and ascorbic acid. In addition, genetic variants more prevalent in South Asians, such as APOE, MTHFR, and SIRT1, may modulate lipid metabolism, methylation, NAD⁺ biosynthesis, and mitochondrial function, warranting further investigation.

Future studies should address these limitations by recruiting larger, multi-centric cohorts with biomarker-confirmed diagnoses, incorporating both plasma and CSF analyses, and adopting longitudinal designs to monitor disease progression. Integration of multi-omics approaches including genomics, proteomics, metabolomics, and microbiomics will be essential to disentangle systemic from CNS-specific changes, elucidate mechanistic pathways, and enable the development of population-tailored biomarkers and therapeutic strategies.

## Conclusion

This study provides the first comprehensive profiling of AD in an Indian cohort, integrating clinical, cognitive, biomarker, and metabolomic data. Key metabolic alterations included elevated leucine, ascorbic acid, and C18:0 dicarboxylic fatty acid, alongside reduced prolinamide and nicotinamide, reflecting disruptions in amino acid metabolism, mitochondrial function, redox balance, and nucleotide biosynthesis-core pathogenic mechanisms of AD.

We also demonstrated strong diagnostic potential for combined plasma biomarkers including pTau181, pTau217, Aβ40, and neurofilament light (NFL) together with metabolic markers such as leucine and C18:0 DCFA, supported by robust AUC values. Correlations between these metabolites and AD biomarkers highlight mechanistic links between metabolic stress and amyloid–tau pathology. Elevated protective metabolites such as eupatilin and ascorbic acid may reflect regional dietary influences, while genetic polymorphisms such as APOE, SIRT1, and MTHFR require further evaluation in relation to these profiles. Nevertheless, the small sample size, cross-sectional design, and lack of external or longitudinal validation limit causal inference and broader applicability. Larger, multi-centric, longitudinal studies with biomarker-confirmed diagnoses are needed to validate these findings. Overall, this study underscores the promise of metabolomics for identifying population-specific biomarkers and advancing personalized diagnostic and therapeutic strategies for AD.

## Supplementary Material

fcaf410_Supplementary_Data

## Data Availability

The data generated and analyzed during study are available from the corresponding author upon reasonable request.
