# Supplementary material for: Comprehensive metabolomics profiling reveals novel biomarkers and pathways for early detection of Alzheimer’s disease
Source: Brain Commun. 2025 Oct 18;7(6):fcaf410. doi: 10.1093/braincomms/fcaf410 (PMC12602867; doi:10.1093/braincomms/fcaf410)

Supplementary Table 1. Differential abundance of plasma metabolites in AD patients

Metabolites	V	T-tests	-Log10(P)	FDR
C18:0,DC FA (Octadecanedicarboxylic acid)	56	5.12E-08	7.2908	2.15E-05
Prolinamide	59	8.12E-08	7.0905	2.15E-05
VALINE	87	3.43E-06	5.4653	0.000511
2-PHOSPHOGLYCERATE	88	3.85E-06	5.4141	0.000511
2-Oxovaleric acid	535	4.86E-06	5.313	0.000516
NICOTINAMIDE	93	6.85E-06	5.1642	0.000581
ETHANOLAMINE PHOSPHATE	531	7.67E-06	5.1153	0.000581
GLYCEROL-MYRISTATE	99	1.33E-05	4.8768	0.00088
C10:3 FA	103	2.03E-05	4.6925	0.001196
LINOLENIC ACID	108	3.39E-05	4.4697	0.001634
Phe-Pro	108	3.39E-05	4.4697	0.001634
7a-Hydroxy-3-oxo-5b-cholanoic acid	111	4.57E-05	4.34	0.002019

C10:3 AC (Decatrenoylcarnitine)	116	7.41E-05	4.1301	0.003022
NICOTINATE	508	8.15E-05	4.089	0.003084
6-Methoxyluteolin	503	0.000129	3.8882	0.004285
methione	503	0.000129	3.8882	0.004285
4-HYDROXY-L-PHENYLGLYCINE	500	0.000169	3.7711	0.004988
C10:3 AC	125	0.000169	3.7711	0.004988
1-O-Hexadecyl-2-O-acetyl-sn-glyceryl-3-phosphorylcholine	499	0.000185	3.7327	0.005163
12,13-Dihydroxy-9Z-octadecenoic acid	128	0.000221	3.6566	0.005565
9-Hydroperoxy-10E,12Z,15Z-octadecatrienoic acid	128	0.000221	3.6566	0.005565
Fumarate	131	0.000285	3.5445	0.006877
ANILINE-2-SULFONATE	493	0.000311	3.5076	0.00716
3-Oxocholic acid	134	0.000367	3.4348	0.00779
NORLEUCINE	134	0.000367	3.4348	0.00779
PC(16:0/0:0)	490	0.000399	3.3987	0.008139
PHOSPHOCHOLINE	489	0.000434	3.363	0.008207

STEARIDONIC ACID	136	0.000434	3.363	0.008207
cis-Aconitic Acid	140	0.000599	3.2224	0.010953
3-Oxostearic acid	142	0.000702	3.1536	0.012005
4-Aminobenzoic acid	142	0.000702	3.1536	0.012005
CIS-8,11,14-EICOSATRIENOIC ACID	481	0.000821	3.0857	0.013595
Eupatilin	145	0.000887	3.0522	0.013823
SPHINGANINE	145	0.000887	3.0522	0.013823
1-Heptadecanoyl-sn-glycero-3-phosphocholine	479	0.000957	3.0189	0.014095
DEOXYCYTIDINE	146	0.000957	3.0189	0.014095
13,14-Dihydro-19(R)-hydroxyprostaglandin E1	148	0.001114	2.953	0.014404
3-METHOXYTYRAMINE	477	0.001114	2.953	0.014404
LEUCINE	477	0.001114	2.953	0.014404
LysoPC(16:1)	477	0.001114	2.953	0.014404
Threitol	148	0.001114	2.953	0.014404
3-Cholic acid	150	0.001294	2.8881	0.01595
ARACHIDONIC ACID	475	0.001294	2.8881	0.01595

Isonicotinic acid	151	0.001393	2.8559	0.016411
lysine	151	0.001393	2.8559	0.016411
3-Methoxybenzenepropanoic acid	473	0.0015	2.824	0.017278
Lysine	472	0.001613	2.7924	0.018189
Caprolactam	155	0.001863	2.7297	0.020154
LysoPC(14:0)	470	0.001863	2.7297	0.020154
Ascorbic acid	467	0.002305	2.6374	0.023948
GUANINE	467	0.002305	2.6374	0.023948
Leucinamide	159	0.002471	2.6071	0.024711
LysoPC(P-16:0)	466	0.002471	2.6071	0.024711
5-AMINOLEVULINATE	465	0.002648	2.577	0.025994
1-Oleoyl-sn-glycero-3-phosphocholine	464	0.002837	2.5471	0.027339
Pipecolic acid	163	0.003251	2.488	0.030767
ASCORBATE	461	0.003477	2.4588	0.031236
DOCOSAHEXAENOATE	461	0.003477	2.4588	0.031236
Glyceraldehyde	164	0.003477	2.4588	0.031236

METHIONINE SULFOXIMINE	460	0.003718	2.4298	0.032838
DIHYDROURACIL	166	0.003973	2.4009	0.034515
Hydroxyoctanoic acid	167	0.004243	2.3723	0.035694
LysoPC(17:0)	458	0.004243	2.3723	0.035694
DECANOATE	457	0.00453	2.3439	0.035831
LysoPC(18:1)	457	0.00453	2.3439	0.035831
PENTADECANOIC ACID	457	0.00453	2.3439	0.035831
XANTHINE	457	0.00453	2.3439	0.035831
PIPECOLIC ACID	456	0.004833	2.3158	0.037671
15-Hydroperoxy-5Z,8Z,11Z,13E-eicosatetraenoic acid	171	0.005495	2.26	0.041607
C20:4,DC FA	171	0.005495	2.26	0.041607
Pentosidine	172	0.005855	2.2324	0.043709
C16:0,OH FA	451	0.006639	2.1779	0.048867
DIETHANOLAMINE	450	0.007064	2.151	0.049917
Methioninesulfoxide	450	0.007064	2.151	0.049917
TRYPTOPHAN	450	0.007064	2.151	0.049917

This table presents results from unpaired two-tailed t-tests comparing metabolite levels between study groups. For each metabolite, the table includes the variable identifier (V), raw p-value, its negative log10 transformation (−Log10(P)), and the Benjamini-Hochberg adjusted False Discovery Rate (FDR). Metabolites with lower FDR values indicate stronger statistical evidence for differential abundance.

Supplementary Table 2. p-value matrix using spearman rank correlation analysis for metabolites versus metabolites in AD patients

P value	C10:3 AC	C10:3 FA	2-PHOSPHOGLYCERATE	VALINE	Prolinamide	C18:0,DC FA (Octadecanedicarboxylic acid)	NICOTINAMIDE	3-Cholic acid	DEOXYCYTIDINE	Threitol	4-Aminobenzoic acid	Glyceraldehyde	Eupatilin	Fumarate	ANILINE-2-SULFONATE	LEUCINE	ASCORBATE	Ascorbic acid	3-Methoxybenzenepropanoic acid	GUANINE
C10:3 AC	NA																			
C10:3 FA	1.00E-10	NA																		
2-PHOSPHOGLYCERATE	0.030889	0.028069	NA																	
VALINE	0.003929	0.009455	4.41E-06	NA																
Prolinamide	0.009455	0.001425	2.47E-05	5.65E-07	NA															
18:0,DC FA (Octadecanedicarboxylic acid	7.39E-05	1.83E-08	0.0013208	0.000602	1.28E-07	NA														
NICOTINAMIDE	0.000624	0.000462	1.53E-05	4.37E-06	1.19E-05	2.93E-05	NA													
3-Cholic acid	0.35381	0.016427	0.086447	0.013353	0.000104	0.001241	0.018588	NA												
DEOXYCYTIDINE	0.000163	0.004181	5.83E-05	0.014837	0.001917	0.005012	1.49E-06	0.70698	NA											
Threitol	0.000644	0.007811	0.00073805	0.002132	0.003173	0.047312	7.44E-05	0.81906	3.84E-14	NA										
4-Aminobenzoic acid	0.15248	0.045914	0.012479	0.000222	1.91E-06	5.91E-06	4.73E-06	0.00204	0.054038	0.13848	NA									
Glyceraldehyde	0.007507	0.078244	0.0026264	0.001735	0.00062	0.004817	0.000394	0.82782	0.00113	0.005226	0.000499	NA								
Eupatilin	0.014021	0.21416	0.017428	0.025046	6.27E-05	0.005665	8.81E-06	0.005166	0.000353	0.007845	0.000462	0.002077	NA							
Fumarate	0.002768	9.56E-05	0.0013136	0.006319	0.000163	1.08E-06	2.08E-06	0.010673	0.002429	0.008469	0.000516	0.068734	0.007261	NA						
ANILINE-2-SULFONATE	0.006508	0.000691	0.046376	6.97E-06	0.001296	0.000571	4.98E-05	0.005796	0.11433	0.13168	1.29E-05	0.005448	0.037888	0.028171	NA					
LEUCINE	0.007408	0.14822	0.0067307	5.38E-05	0.007845	0.087213	0.008323	0.067889	0.012582	0.043659	0.25997	0.008979	0.10427	0.42218	0.11512	NA				
ASCORBATE	0.05121	0.003709	0.00033661	0.038882	0.004773	0.002881	0.000306	0.057634	0.079427	0.21217	0.019473	0.15744	0.12311	5.76E-05	0.021268	0.48739	NA			
Ascorbic acid	0.039897	0.006837	0.0037448	0.016656	0.005652	0.000691	0.000354	0.006581	0.24375	0.38417	0.006305	0.16855	0.13362	0.000163	0.013936	0.11964	0	NA		
3-Methoxybenzenepropanoic acid	0.055186	0.022259	0.0017907	0.002429	0.001	0.02812	0.029424	0.23967	0.045837	0.054566	0.13648	0.026522	0.26054	0.19016	0.043586	0.000358	0.033053	0.019661	NA	
GUANINE	0.085307	0.077307	0.009374	0.081108	0.007181	0.062396	0.033821	0.84904	0.036596	0.24622	0.18787	0.001069	0.2454	0.36187	0.386	0.38747	0.016689	0.029212	0.14142	NA

This table summarizes p-value for Pearson rank correlations coefficient among plasma metabolites, highlighting modular biochemical relationships. Positive correlations indicate co-regulation or shared metabolic pathways, while negative correlations suggest inverse regulation.

Supplementary Table 3. p-value matrix using Spearman rank correlation coefficients for plasma biomarkers versus biomarkers in AD patients

	Aβ 40	Aβ 42	Aβ 42/40 ratio	pTau181	pTau217	pTau217/Aβ42	Glutamate	8OHDG	BDNF	CRP	NFL	APOE	APOE4
**Aβ 40**	NA												
**Aβ 42**	0.641	NA											
**Aβ 42/40 ratio**	0.001	0.000	NA										
**pTau181**	0.863	0.547	0.818	NA									
**pTau217**	0.487	0.383	0.268	0.897	NA								
**pTau217/Aβ42**	0.202	0.010	0.258	0.983	0.000	NA							
**Glutamate**	0.067	0.168	0.674	0.015	0.133	0.009	NA						
**8OHDG**	0.487	0.383	0.268	0.897	0.000	0.000	0.133	NA					
**BDNF**	0.680	0.312	0.326	0.929	0.116	0.448	0.628	0.116	NA				
**CRP**	0.563	0.656	0.400	0.923	0.075	0.132	0.501	0.075	0.334	NA			
**NFL**	0.328	0.167	0.748	0.108	0.919	0.420	0.373	0.919	0.226	0.838	NA		
**APOE**	0.222	0.477	0.141	0.106	0.702	0.750	0.293	0.702	0.700	0.539	0.877	NA	
**APOE4**	0.220	0.927	0.365	0.895	0.479	0.888	0.790	0.479	0.991	0.458	0.599	0.503	NA

This table summarizes the corresponding *p*-value among plasma biomarkers in Alzheimer's disease (AD). Spearman *p*-values denote the strength and significance of pairwise correlations between biomarkers versus biomarkers. Biomarkers include amyloid-beta (Aβ40, Aβ42, Aβ42/40 ratio), tau isoforms (pTau181, pTau217, pTau217/Aβ42), metabolic and stress markers (glutamate, 8OHdG, BDNF, CRP, NFL), and genetic risk factors (APOE, APOE4).

Supplementary Table-4. p-value matrix using spearman rank correlations coefficient for metabolites versus biomarkers in AD patients

	2-PHOSPHOGLYCERATE	3-Cholic acid	3-Methoxybenzenepropanoic acid	4-Aminobenzoic acid	ANILINE-2-SULFONATE	ASCORBATE	Ascorbic acid	C10:3 AC	C10:3 FA	C18:0,DC FA (Octadecanedicarboxylic acid)	DEOXYCYTIDINE	Eupatilin	Fumarate	Glyceraldehyde	GUANINE	LEUCINE	NICOTINAMIDE	Prolinamide	Threitol	VALINE
**Aβ 40**	0.479	0.550	0.441	0.106	0.534	0.171	0.760	0.578	0.493	0.056	0.919	0.188	0.444	0.686	0.253	0.148	0.228	0.070	0.855	0.126
**Aβ 42**	0.443	0.423	0.287	0.215	0.064	0.900	0.984	0.037	0.888	0.926	0.794	0.846	0.588	0.457	0.657	0.093	0.103	0.093	0.639	0.874
**Aβ 42/40 ratio**	0.150	0.688	0.270	0.057	0.650	0.997	0.621	0.095	0.864	0.212	0.939	0.500	0.968	0.619	0.593	0.512	0.089	0.049	0.707	0.734
**pTau181**	0.159	0.968	0.321	0.438	0.476	0.211	0.347	0.564	0.875	0.959	0.846	0.914	0.428	0.512	0.937	0.745	0.865	0.465	0.756	0.033
**pTau217**	0.273	0.171	0.521	0.484	0.429	0.945	0.808	0.154	0.553	0.353	0.724	0.006	0.864	0.858	0.181	0.539	0.456	0.719	0.885	0.233
**pTau217/Aβ42**	0.974	0.098	0.333	0.193	0.575	0.482	0.479	0.990	0.869	0.411	0.878	0.003	0.515	0.974	0.270	0.411	0.743	0.304	0.365	0.618
**Glutamate**	0.245	0.558	0.110	0.169	0.538	0.301	0.737	0.974	0.648	0.166	0.253	0.098	0.685	0.653	0.591	0.436	0.601	0.389	0.049	0.194
**8OHDG**	0.273	0.171	0.521	0.484	0.429	0.945	0.808	0.154	0.553	0.353	0.724	0.006	0.864	0.858	0.181	0.539	0.456	0.719	0.885	0.233
**BDNF**	0.870	0.274	0.744	0.911	0.727	0.137	0.096	0.717	0.233	0.086	0.488	0.913	0.435	0.010	0.611	0.885	0.492	0.475	0.956	0.659
**CRP**	0.628	0.876	0.802	0.064	0.322	0.623	0.596	0.901	0.076	0.220	0.763	0.307	0.290	0.510	0.777	0.853	0.865	0.473	0.778	0.732
**NFL**	0.923	0.163	0.566	0.370	0.601	0.708	0.655	0.262	0.737	0.466	0.320	0.199	0.909	0.275	0.848	0.099	0.625	0.630	0.619	0.753
**APOE**	0.476	0.214	0.299	0.974	0.160	0.262	0.940	0.707	0.501	0.188	0.880	0.318	0.051	0.607	0.025	0.735	0.615	0.382	0.872	0.471
**APOE4**	0.680	0.507	0.648	0.653	0.332	0.881	0.656	0.381	0.729	0.243	0.756	0.881	0.896	0.781	0.690	0.080	0.878	0.270	0.959	0.712

This table presents p-value for associations between 20 -metabolites and key plasma biomarkers, including amyloid beta (Aβ40, Aβ42, Aβ42/40 ratio), tau phosphorylation markers (pTau181, pTau217, pTau217/Aβ42), neuroinflammatory and oxidative stress indicators (CRP, BDNF, 8OHdG, Glutamate), neurodegeneration markers (NFL), and genetic risk factors (APOE, APOE4). Positive r values indicate direct associations, while negative values suggest inverse relationships. Statistically significant correlations ($p < 0.05$) are highlighted to emphasize potential mechanistic relevance.

Supplementary figure legend:

Supplementary Figure 1. Study design and workflow for biomarker and metabolomics analysis in Alzheimer's disease (AD) and healthy controls (HCs). A total of 50 participants (n = 25 AD subjects, n = 25 age-matched HCs) were recruited following defined inclusion and exclusion criteria. Clinical diagnosis of AD was based on the National Institute on Aging–Alzheimer's Association (NIA-AA) guidelines, supported by cognitive assessments [Addenbrooke's Cognitive Examination III (ACE-III), Clinical Dementia Rating–Global score (CDR-G)], and confirmed by magnetic resonance imaging (MRI) and fluorodeoxyglucose-positron emission tomography (FDG-PET) imaging. Blood samples were collected from all participants, and plasma was isolated for biomarker analysis and untargeted metabolomics using liquid chromatography–mass spectrometry (LC-MS). Biomarker assessment included measurement of plasma levels of AD-related markers such as amyloid-β40 (Aβ40), amyloid-β42 (Aβ42), Aβ42/40 ratio, phosphorylated tau species (pTau217, pTau181), C-reactive protein (CRP), 8-hydroxy-2′-deoxyguanosine (8-OHdG), glutamate, brain-derived neurotrophic factor (BDNF), apolipoprotein E (APOE), and APOE4 genotype. LC-MS–based untargeted metabolomics was used for identification and quantification of metabolites. Data were analyzed through quality control, normalization, statistical and pathway enrichment analyses, and correlation of biomarker levels with metabolomics profiles.

Flow diagram

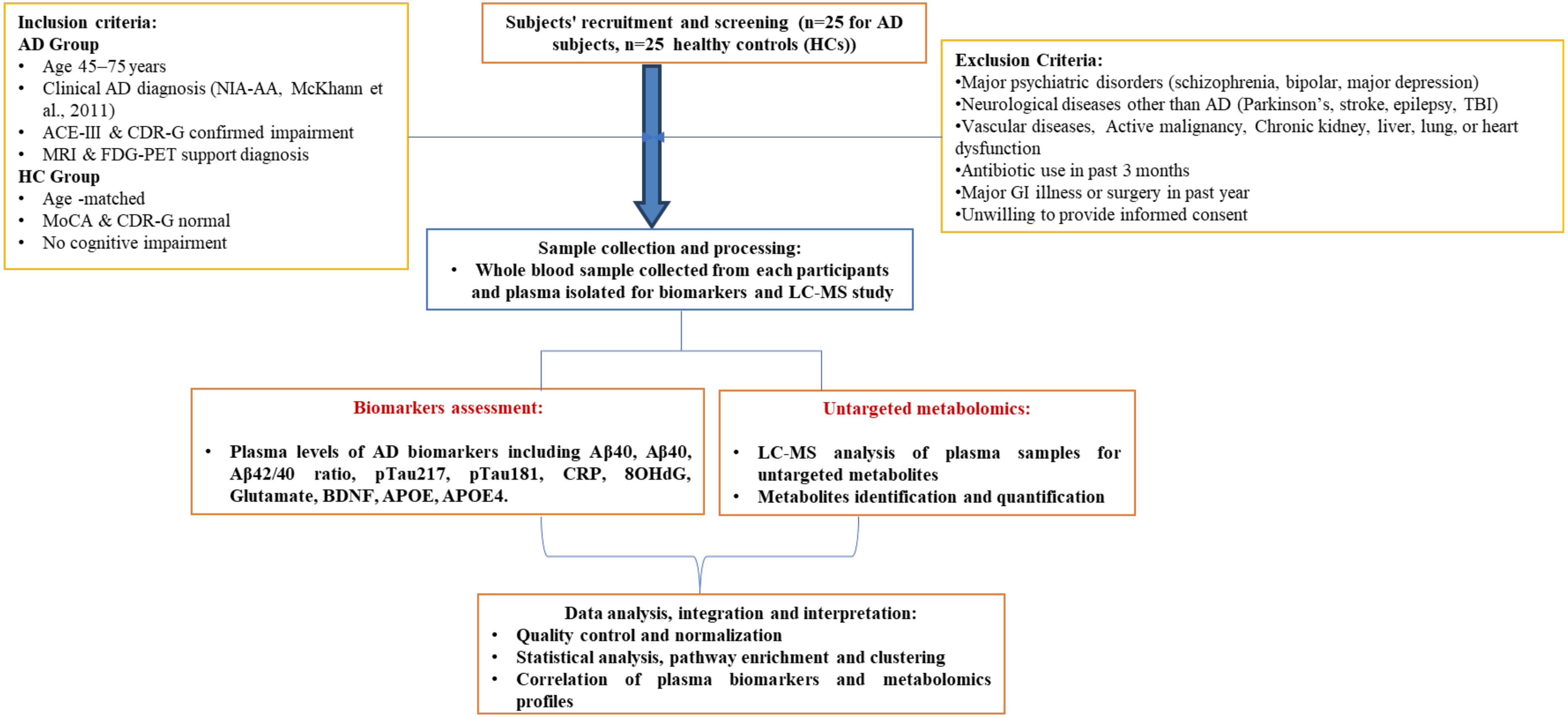

Supplementary Figure 2. Statistical analysis and heatmap of differential metabolites between Alzheimer's disease (AD) and healthy controls (HC)

Supplementary Figure 2A. T-test results comparing Alzheimer's disease (AD) and healthy control (HC) groups. Each datapoint represents an individual metabolite identified through untargeted metabolomic profiling. The analysis reveals 75 metabolites with significant differences between AD and HC groups ($p < 0.05$) and 455 metabolites without significant differences ($p \geq 0.05$). Colors indicate the –log10(FDR-adjusted p-values), with higher values reflecting stronger significance. Experimental units: AD ($n = 25$) and HC ($n = 25$). This analysis highlights metabolic alterations associated with AD, which may serve as potential biomarkers.

Supplementary Figure 2B. Hierarchical clustering heatmap of the top 50 metabolites differentiating Alzheimer's disease (AD) from healthy controls (HC). Rows represent metabolites, and columns represent individual samples. Each datapoint represents the relative abundance (log_2-normalized intensity) of a metabolite. Data source: normalized data; standardization: autoscale features. Statistical comparisons between AD and HC groups were performed using Student's *t*-test ($p < 0.05$). Clustering was conducted using Euclidean distance and complete linkage. Experimental units: AD ($n = 25$) and HC ($n = 25$). The color scale (log_2 intensity values) is displayed using the Plasma (blue–magenta), with blue indicating lower and magenta indicating higher metabolite levels. This visualization highlights distinct metabolomic profiles between AD and HC groups, aiding in the identification of potential biomarkers.

Supplementary Figure-2A.

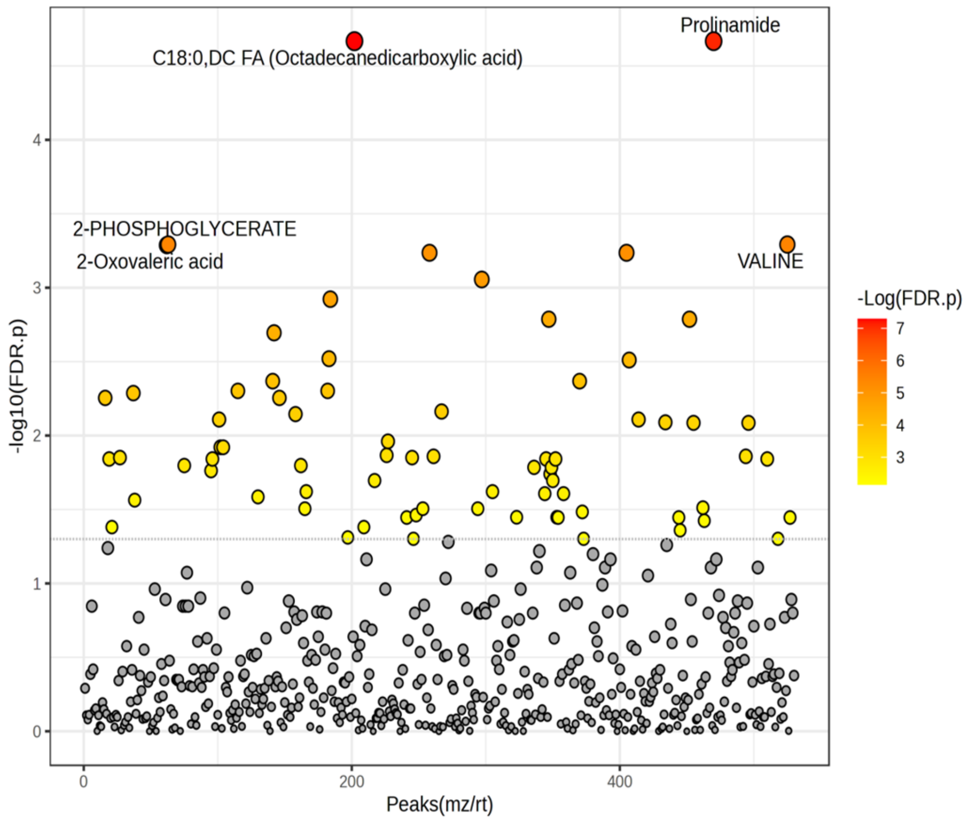

Supplementary Figure 2B.

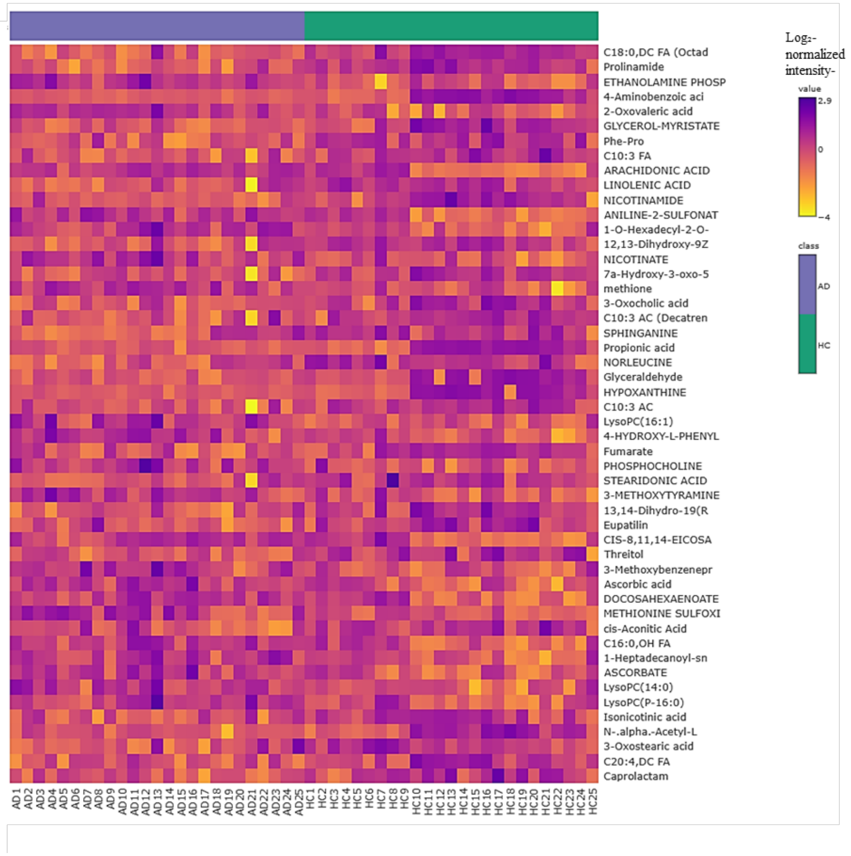

Supplementary Figure 3. Cross-correlation analysis of significant metabolites in Alzheimer's disease (AD). The heatmap depicts pairwise correlations of key upregulated and downregulated metabolites identified from the volcano plot comparison between Alzheimer's disease (AD) and Healthy Controls (HC). Correlations were calculated using Spearman correlation coefficient test. The experimental unit corresponds to individual plasma samples (AD: n = 25; HC: n = 25). The color scale, labeled "Correlation coefficient (r)", ranges from –1 (perfect negative correlation) to +1 (perfect positive correlation). Warm colors (red) indicate positive correlations, while cool colors (blue) indicate negative correlations, with intensity reflecting correlation strength. Statistical significance is denoted as: *$p < 0.05$, **$p < 0.01$, ***$p < 0.001$, ****$p < 0.0001$, and ns = not significant.

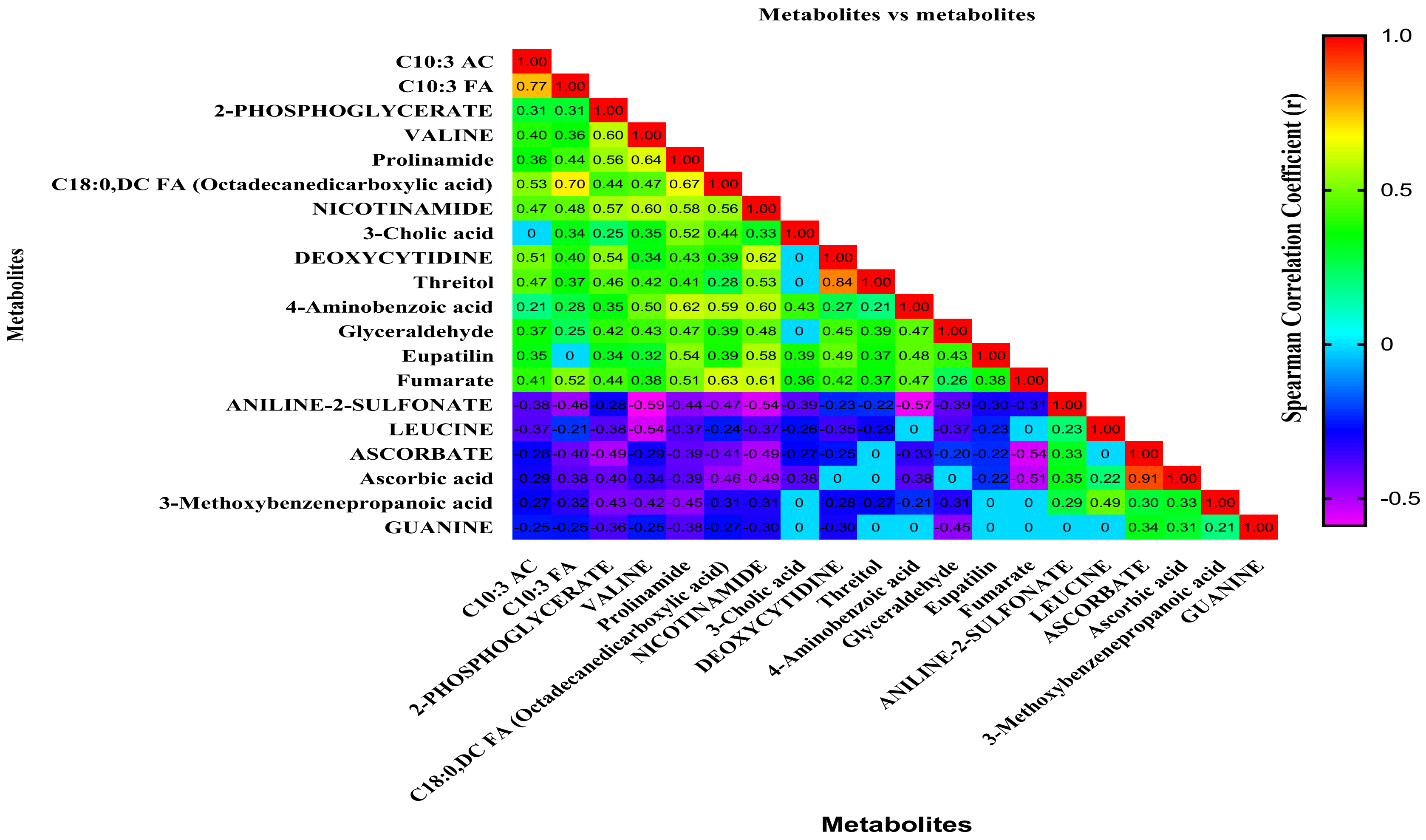
Metabolites vs metabolites
Metabolites
C10:3 AC 1.00
C10:3 FA 0.77 1.00
2-PHOSPHOGLYCERATE 0.31 0.31 1.00
VALINE 0.40 0.36 0.60 1.00
Prolinamide 0.36 0.44 0.56 0.64 1.00
C18:0,DC FA (Octadecanedicarboxylic acid) 0.53 0.70 0.44 0.47 0.67 1.00
NICOTINAMIDE 0.47 0.48 0.57 0.60 0.58 0.56 1.00
3-Cholic acid 0 0.34 0.25 0.35 0.52 0.44 0.33 1.00
DEOXYCYTIDINE 0.51 0.40 0.54 0.34 0.43 0.39 0.62 0 1.00
Threitol 0.47 0.37 0.46 0.42 0.41 0.28 0.53 0 0.84 1.00
4-Aminobenzoic acid 0.21 0.28 0.35 0.50 0.62 0.59 0.60 0.43 0.27 0.21 1.00
Glyceraldehyde 0.37 0.25 0.42 0.43 0.47 0.39 0.48 0 0.45 0.39 0.47 1.00
Eupatilin 0.35 0 0.34 0.32 0.54 0.39 0.58 0.39 0.49 0.37 0.48 0.43 1.00
Fumarate 0.41 0.52 0.44 0.38 0.51 0.63 0.61 0.36 0.42 0.37 0.47 0.26 0.38 1.00
ANILINE-2-SULFONATE -0.38 -0.46 -0.28 -0.59 -0.44 -0.47 -0.54 -0.39 -0.23 -0.22 -0.57 -0.39 -0.30 -0.31 1.00
LEUCINE -0.37 -0.21 -0.38 -0.54 -0.37 -0.24 -0.37 -0.26 -0.35 -0.29 0 -0.37 -0.23 0 0.23 1.00
ASCORBATE -0.28 -0.40 -0.49 -0.29 -0.39 -0.41 -0.49 -0.27 -0.25 0 -0.33 -0.20 -0.22 -0.54 0.33 0 1.00
Ascorbic acid -0.29 -0.38 -0.40 -0.34 -0.39 -0.46 -0.49 -0.38 0 0 -0.38 0 -0.22 -0.51 0.35 0.22 0.91 1.00
3-Methoxybenzenepropanoic acid -0.27 -0.32 -0.43 -0.42 -0.45 -0.31 -0.31 0 -0.28 -0.27 -0.21 -0.31 0 0 0.29 0.49 0.30 0.33 1.00
GUANINE -0.25 -0.25 -0.36 -0.25 -0.38 -0.27 -0.30 0 -0.30 0 0 -0.45 0 0 0 0 0.34 0.31 0.21 1.00
C10:3 AC
C10:3 FA
2-PHOSPHOGLYCERATE
VALINE
Prolinamide
C18:0,DC FA (Octadecanedicarboxylic acid)
NICOTINAMIDE
3-Cholic acid
DEOXYCYTIDINE
Threitol
4-Aminobenzoic acid
Glyceraldehyde
Eupatilin
Fumarate
ANILINE-2-SULFONATE
LEUCINE
ASCORBATE
Ascorbic acid
3-Methoxybenzenepropanoic acid
GUANINE
Metabolites
Spearman Correlation Coefficient (r)
1.0
0.5
0
-0.5

Supplementary Figure 4. ROC curve and cross-correlation analysis of plasma biomarkers in Alzheimer's disease (AD).

Supplementary Figure 4A. The ROC curve evaluates the diagnostic performance of plasma biomarkers in distinguishing AD patients from Healthy Controls (HC). Sensitivity (true positive rate) is plotted against 1-specificity (false positive rate) for each biomarker. The area under the curve (AUC) quantifies diagnostic accuracy: Aβ40 (AUC = 0.7840), Aβ42 (AUC = 0.5216), Aβ42/40 ratio (AUC = 0.7512), pTau181 (AUC = 0.9784), pTau217 (AUC = 0.7632), pTau217/Aβ42 ratio (AUC = 0.7344), BDNF (AUC = 0.9760), NFL (AUC = 0.7480), CRP (AUC = 0.8040), 8OHdG (AUC = 0.7032), APOE (AUC = 0.5136), and APOE4 (AUC = 0.6304). A higher AUC indicates greater discriminatory power between AD and HC groups.

Supplementary Figure 4B. Cross-correlation analysis of AD plasma biomarkers. Relationships between key plasma biomarkers in AD were assessed using the Spearman rank correlation test. The experimental unit (N) corresponds to individual plasma samples (AD: n = 25; HC: n = 25). The correlation coefficients (r) are represented by a color scale titled "Correlation coefficient (r)", ranging from –1 (perfect negative) to +1 (perfect positive), with red indicating positive correlations and blue indicating negative correlations; the intensity of the color reflects the strength of the correlation. Statistical significance is indicated as *$p < 0.05$, **$p < 0.01$, ***$p < 0.001$, ****$p < 0.0001$; ns = not significant. This analysis highlights the interrelationships of AD biomarkers and their combined role in AD pathology.

Figure 4A.

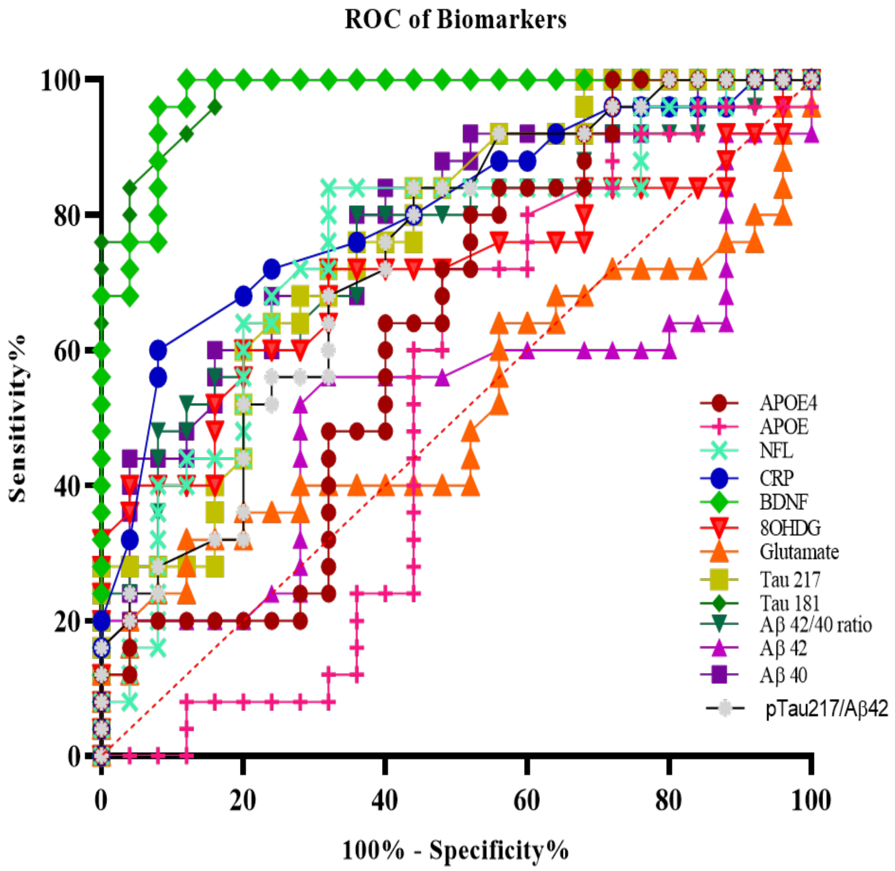

Figure 4B.

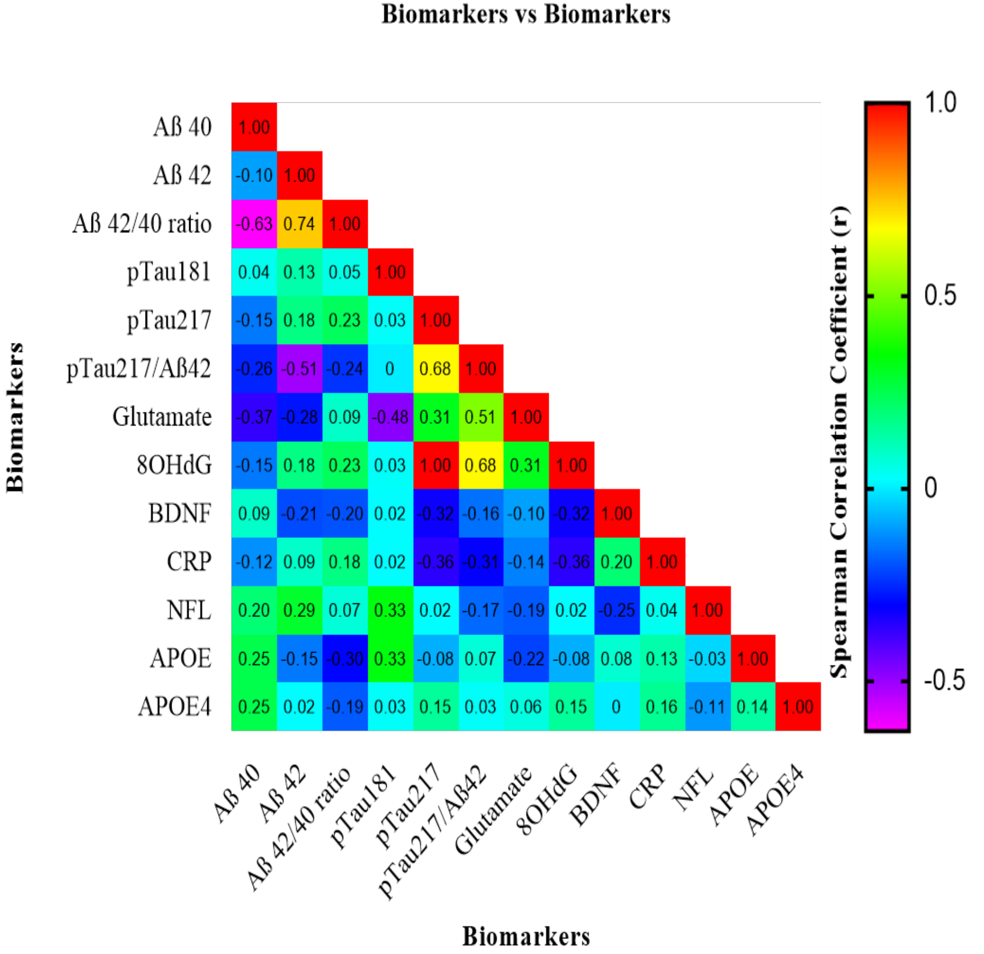

Supplement: fcaf410_Supplementary_Data [file fcaf410_supplementary_data.pdf]
